# Interfacial SnS/SnO_*x*_ heterostructures on GO–PVP enable the removal of methylene blue and enhanced redox catalysis

**DOI:** 10.1039/d6ra02696f

**Published:** 2026-07-06

**Authors:** Ashlesha P. Kawale, Nishant Shekhar, Kumari Anchal, S. Y. Bodkhe, Subhash Banerjee, Arti Srivastava

**Affiliations:** a Department of Chemistry, Guru Ghasidas Vishwavidyalaya Koni Bilaspur-495009 CG India reach2arti@yahoo.co.uk; b National Environmental Engineering Research Institute, NEERI Nagpur-440020 MH India

## Abstract

Here, we demonstrate the synthesis of SnS/SnO_*x*_ nanoparticles (NPs) and a ternary SnS/SnO_*x*_–GO–PVP hybrid nanocomposite (NC) *via* a hydrothermal method for multimodal applications. SnS/SnO_*x*_–GO–PVP NC was characterized by FT-IR spectroscopy, powder XRD, SEM, XPS, BET, and TGA studies. Owing to its higher surface area, surface functionalities, and interfacial heterostructure, SnS/SnO_*x*_–GO–PVP NC exhibited superior adsorption of methylene blue (98% removal within 100 min at 4–10 ppm). Furthermore, SnS/SnO_*x*_–GO–PVP NC exhibited remarkable catalytic activities in organic redox reactions, facilitating the selective reduction of nitrobenzene and its derivatives to the corresponding azobenzenes in yields ranging from 70% to 83% and the oxidation of alcohols to the corresponding aldehydes and ketones in good to excellent yields (75–87%). The catalyst was reused for up to five cycles without any appreciable loss in catalytic activity. These results demonstrate that SnS/SnO_*x*_–GO–PVP NC is a robust, cost-effective, and multifunctional catalyst for dye remediation and redox catalysis.

## Abstract 2

Hydrothermally synthesized SnS/SnO_*x*_–GO–PVP nanocomposite exhibited 98% methylene blue removal, catalyzed nitro reduction and alcohol oxidation with high yields, and remained reusable for five cycles as a multifunctional catalyst.

## Graphical abstract

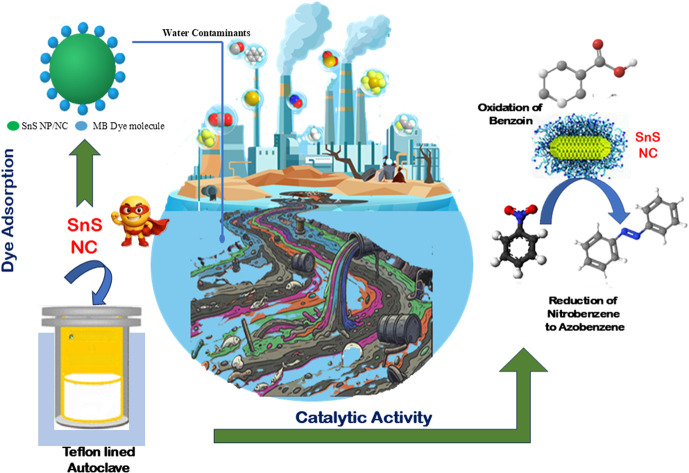

## 1. Introduction

Industrial expansion has endangered the lives of people and caused environmental hazards. Industries like textiles, dyeing, paints, leather, and pigments produce large quantities of dye-containing effluent.^1^ Used to enhance the visual and useful properties of many products, these chemical colors are brightly coloured and complex. But they will be harmful if they are not treated and released into the ecosystem.^2^ These things can hurt sea creatures in the water. They reduce sunlight penetration into water bodies, thereby inhibiting photosynthesis in aquatic plants. Some may harm human and animal health by causing cancer, mutations, and allergies. Their persistence in nature worsens ecological and health threats, because they resist degradation through natural processes.^3^ Methylene blue (MB), an example of positively charged dyes, is highly harmful. Because they bind to negative membrane surfaces, they enter cells rapidly and accumulate within cells. This mechanism leads to severe biological damage.^4,5^ For local and rural firms with limited funds, effective solutions are expected to be practically and financially feasible.^6^ Current fabric waste cleaning systems regularly employ a combination of mechanical, chemical, and bio-based processes such as flotation, adsorption, coagulation, Fenton oxidation, ozonation, fungal treatment, and microbial breakdown.^7^ All of these methods can be advantageous because of the high efficiencies in dye removal or the selective targeting process. Similarly, they also have some drawbacks like a high cost of energy, harmful by-products production, and difficulty in the application on a small scale.^8^ Biological treatments, for example, are slow and less effective for persistent dyes, whereas chemical treatments such as Fenton produce residual sludge, necessitating additional handling and disposal processes.^9^

Surface binding stands out among these methods because it is simple, effective, does not produce toxic compounds, and is inexpensive – all of which are very important when resources are limited.^10^ This approach attracts dye particles to the outer layer of an absorbent material, which may be engineered to trap dyes more effectively while facilitating recovery.^11^ Polymeric nanocomposites, metal–organic frameworks (MOFs),^12^ zeolites, activated carbon, and semiconductor-based nanomaterials are among the new alternatives made possible by advancements in nanoscale research. These compounds stand out owing to their broad exposure^12^ zones and tunable chemical properties, which enhance their potential to remove pigments from contaminated water.^12^ Investigating such new techniques is critical when trying to replace obsolete approaches with cleaner, stronger purification systems.^13,14^ Narrow-bandgap metal sulfides are currently extensively studied for dye adsorption. Their appeal derives from various electrical and chemical properties, such as high light absorption, tunable energy gaps, or activity under visible light exposure.^14,15^ Tin(ii) sulfide (SnS), a member of the IV–VI semiconductor family, stands out in pollution management efforts due to its high visible-range absorption and direct bandgap spanning between 1.3 and 2.33 eV.^16^ Such features allow SnS to successfully bind organic dyes while also driving photo-assisted removal processes under light. SnS is an environmentally conscientious alternative to traditional materials like activated carbon or titanium dioxide, which are frequently associated with costly or harmful environmental activities.^17^ SnS's chemical stability in aqueous solutions ensures consistent, long-term performance in wastewater treatment applications, while its orthorhombic layered crystal structure increases surface area and provides a large number of active sites for dye adsorption.^18^ According to studies, this molecule successfully eliminates a wide range of organic contaminants, notably positively charged colors like methylene blue dye, by non-chemical attachment processes.^19^ SnS stands out as a practical option for addressing important issues related to colored effluents from manufacturing processes because of its adaptability, excellent performance, and low environmental impact.^20^ Researchers altered the surfaces of SnS to make it more resilient over time, more liquid-spreading, and more attractive to contaminants, all of which improved the removal of pollutants. One effective technique is to employ polyvinylpyrrolidone (PVP), a high molecular weight polymer that is commonly used as a stabilizing agent and surfactant in the fabrication of nanomaterials due to its non-toxic, biocompatible, and stabilizing qualities. PVP's amphiphilic structure prevents SnS/SnO_*x*_ Nps from clumping together and enhances their interaction with dye molecules by possessing a hydrophilic pyrrolidone group and a hydrophobic alkyl backbone. Additionally, PVP makes it easier to synthesize SnS/SnO_*x*_–GO–PVP NCs, ensuring regular particle sizes and surface characteristics crucial elements for achieving high adsorption efficacy. It also helps in efficient adsorbent design because of its capacity to produce hydrogen bonds and electrostatic interactions with dyes, which increase selectivity and efficiency.^21^

Graphene oxide (GO), a two-dimensional carbon material, has received attention as a useful component in NC adsorbents due to its excellent mechanical stability, large surface area, and abundant oxygen-based groups such as hydroxyl, carboxyl, and epoxy.^22^ These groups operate as critical binding locations, allowing GO to trap dye molecules using hydrogen bonds, electrostatic interactions, or π–π stacking.^23^ When dealing with aromatic dyes like MB, such interactions are very effective.^24^ Because of its broad surface coverage and adjustable surface characteristics, GO works well with other adsorbents, improving both the uptake performance and the reuse potential of hybrid systems. When coupled with SnS/SnO_*x*_, it forms a solid structure that aids in the distribution of NPs, enhancing access to active regions and improving dye removal efficacy.^25^

The production of an SnS/SnO_*x*_ NC with PVP and GO is one method for developing materials that can effectively remove pollutants from wastewater. When combined, they are more effective in targeting particular colors, adsorbing pollutants, and being reused than when used independently.^26^ This NC can be manufactured by a simple, scalable hydrothermal technique. Using SnS/SnO_*x*_, GO, and PVP results in a NC with an improved surface structure and more accessible active sites. As a result, MB dye is collected more efficiently, making it a feasible treatment option for polluted water. The reaction environment, interfacial interactions, and surface chemistry have a significant impact on catalytic performance.^27^ In addition to binding contaminants, this material promotes redox reactions due to the SnS/SnO_*x*_ framework, which accelerates electron transport and stabilizes unstable reaction intermediates. Consequently, it facilitates the conversion of nitrobenzene to azobenzene and of alcohols to carbonyls, demonstrating its dual utility in both water purification and organic catalysis.^28^

## 2. Experimental

### 2.1. Materials

Sodium sulphide hydrate (Na_2_S·9H_2_O), tin chloride (SnCl_2_), ethylene glycol, graphite powder, sodium nitrate (NaNO_3_), potassium permanganate (KMnO_4_), concentrated sulphuric acid, 30% hydrogen peroxide (H_2_O_2_), polyvinylpyrrolidone (PVP), methylene blue, nitrobenzene, sodium borohydride (NaBH_4_), methanol, ethyl acetate, brine solution, petroleum ether, benzoin, and toluene were used. Methylene blue is a cationic dye used in many industries as a colouring reagent. It was purchased from SCI Fine Chemicals and used without any further modification. All the chemicals and solvents were purchased from Sigma-Aldrich and used as received for synthesis and analysis.

### 2.2. Instrumentation

A quartz cuvette with a width of 10 mm was used to record UV-visible spectra on a Shimadzu UV 1800 spectrophotometer. Using KBr pellets as the backdrop matrix and Fourier transform infrared (FTIR) spectroscopy (JASCO TOKYO-4700), the functional groups were found by scanning in the wavenumber range of 4000–500 cm^−1^. The crystal structure and phase were investigated using powder X-ray diffraction (XRD) on a Rigaku diffractometer equipped with a Cu rotating anode source (18 kW). The 2*θ* range from 10° to 80° and a scanning speed of 2° min^−1^ were used to collect the data. To ascertain thermal stability, thermogravimetric analysis (TGA) was carried out using a METTLER TOLEDO analyzer. Thermogravimetric analysis was performed under a nitrogen atmosphere over the temperature range 50–600 °C at a rate of 10 °C every minute. The chemical compositions and structural features of the samples were ascertained using nuclear magnetic resonance (NMR) spectroscopy. Using CDCl_3_ as a solvent, the ^1^H and ^13^C NMR spectra were acquired at 500 MHz and 20 °C using a JEOL AL 500 FT-NMR spectrometer. A SCIEX X500R QTOF equipment was used for mass spectrometry to verify the molecular mass of the components. Polystyrene standards with both narrow and wide molecular weight distributions were used to calibrate a T6000M mixed-bed column. A GEMINI 560 Carl Zeiss, which had a high-resolution FESEM (with EDS and EBSD), was also used for morphological characterization. X-ray photoelectron spectroscopy (XPS) on a PHI 5000 VersaProbe III system was used to analyze the surface chemical composition. The C 1s peak at 284.7 eV was used to calibrate the binding energy. The Barrett–Joyner–Halenda (BJH) technique was used to calculate the sample's surface area and average pore size. Volumetric gas adsorption studies were performed using a Brunauer–Emmett–Teller (BET) surface analyzer (Quantachrome Instruments, USA, model: Autosorb iQ2) in the pressure range of 0–900 mmHg to measure nitrogen adsorption/desorption at 77.4 K. The samples were degassed at 150 °C for an entire night before the tests in order to remove any gases from the environment, especially bound moisture. All the measurements were performed on the samples in powder form.

### 2.3. Synthesis of graphene oxide (GO)

GO was prepared from a graphite powder using a modified Hummer's process.^29^ Briefly, a round-bottom flask was filled with 0.5 g of sodium nitrate and 1 g of the graphite powder. After that, 23 mL of concentrated sulfuric acid was added while the liquid was slowly agitated. After 1 hour of stirring, 3 g of potassium permanganate was added gradually, keeping the temperature below 20 °C to prevent overheating or explosions. The mixture was then heated to 35 °C and shaken for 8 hours. The mixture was diluted with 500 mL of distilled water while being vigorously stirred. After that, 5 mL of 30% hydrogen peroxide was added to ensure the potassium permanganate reaction was complete. The resulting mixture was cleaned with 0.1 M hydrochloric acid and then washed with distilled water. After filtration and drying, graphene oxide was obtained as a black solid.

### 2.4. Synthesis of SnS/SnO_*x*_ NPs

**Fig. 1:**
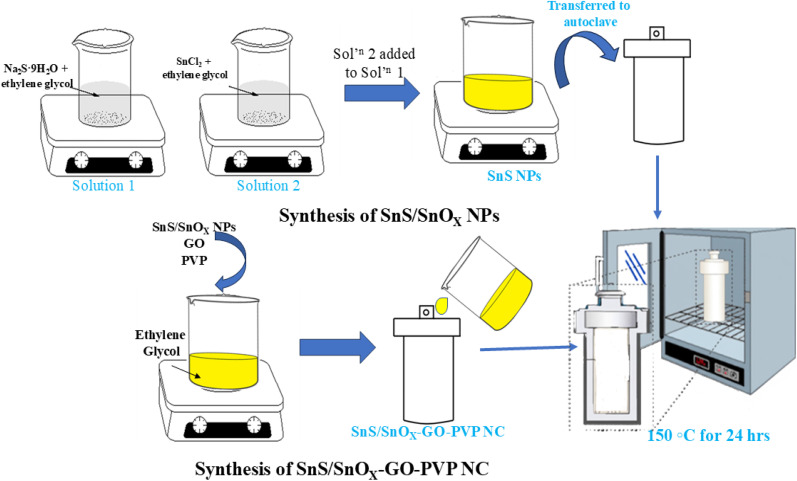
Schematic of the stepwise synthesis of SnS/SnO_*x*_ NPs and SnS/SnO_*x*_–GO–PVP NC.

Typically, 0.36 g of sodium sulphide hydrate was mixed with 80 mL of ethylene glycol, and 0.33 g of tin chloride was dissolved separately in 30 mL of ethylene glycol, as depicted in Fig. 1. Both reaction mixtures were added dropwise at temperatures above 60 °C. The sodium sulphide hydrate solution was then gradually stirred while the tin chloride solution was added dropwise. As the two liquids combined, the initially clear mixture gradually became black. After that, the mixture was transferred to a Teflon-coated stainless-steel autoclave. After being heated to 150 °C in an oven and maintained there for 24 hours, the autoclave was spontaneously cooled to room temperature. The obtained black solid was collected by centrifugation and repeatedly washed with distilled water and ethanol. The obtained black precipitate was oven-dried for 4 hours at 60 °C.^30^

### 2.5. Synthesis of SnS/SnO_*x*_–GO–PVP NC

To synthesize the tin precursor solution, 1.0 g of tin chloride was mixed with 20 mL of ethylene glycol, stirred using a magnetic stirrer, and heated to 80 °C. 1.07 g of sodium sulphide hydrate was dissolved in 20 mL of ethylene glycol in a separate beaker. Meanwhile, 10 mL of distilled water was used to disperse 20 mg of GO, which was then sonicated for 30 minutes. Following sonication, 40 mg of PVP was added to the GO mixture and fully dissolved with stirring. The GO–PVP solution was added dropwise to the tin precursor solution while vigorously stirring. The sodium sulphide hydrate solution was gradually added as well. As these solutions were mixed, the reaction mixture's color turned dark brown. Stirring was continued for an additional 30 minutes at 80 °C. The resulting mixture was poured into a 100 mL Teflon-lined stainless-steel autoclave, sealed, and heated at 150 °C for 12 hours, as shown in Fig. 1. Following cooling, the black-colored product was separated by centrifuging it at 5000 rpm for 5 minutes. It was cleaned many times using ethanol and distilled water before being oven-dried for 4 hours at 70 °C.

### 2.6. Adsorption studies

SnS/SnO_*x*_ NP and SnS/SnO_*x*_–GO–PVP NC batch adsorption experiments were conducted at room temperature. A stock solution of MB dye (20 mg L^−1^ or 20 ppm) was prepared by dissolving 5 mg of MB dye in 250 mL of distilled water to examine the impact of the initial dye concentration. To prepare all subsequent solutions, the stock solution was diluted. A direct correlation between the absorbance and dye concentration was discovered. The dye adsorption experiment proceeded by immersing 5 mg of the as-synthesised SnS/SnO_*x*_ NPs and SnS/SnO_*x*_–GO–PVP NC in 10 mL of a MB solution at varying dye solution concentrations (4, 6, 8, and 10 ppm). The parameters were studied, including contact time and initial dye concentration. The adsorption of the remaining dye concentrations was assessed by measuring the absorbance at *λ*_max_ = 664 nm. The adsorption capacities of SnS/SnO_*x*_ NPs and SnS/SnO_*x*_–GO–PVP NC for MB at adsorption time *Q*_*t*_ and equilibrium time *Q*_e_ were calculated using eqn (1) and (2), respectively. The removal efficiency (RE) of MB was determined using eqn (3).1
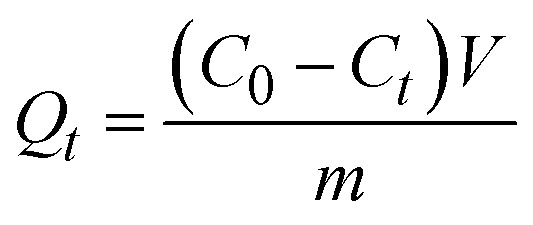
2
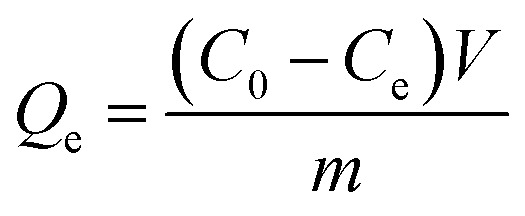
3
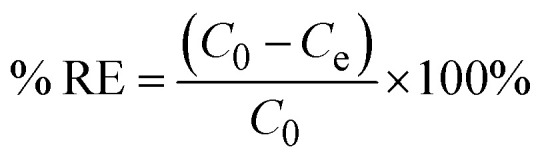


#### 2.6.1. Optimization of variables

To optimize MB dye removal utilizing SnS/SnO_*x*_ NPs and SnS/SnO_*x*_–GO–PVP NC, the adsorption conditions were tuned to operate near the intrinsic capacity of each material. The contact time, initial dye concentration, and pH of the solution were considered critical factors because they influence the mass-transfer driving force and the progression to equilibrium. Most dye-adsorbent systems exhibit fast absorption in the early stages and progressively slow down when equilibrium is approached, with the overall behavior often fitting a pseudo-second-order kinetic model, indicating chemisorption.^31^ Increasing the initial dye concentration typically improves the adsorption capacity by increasing the concentration gradient, but extremely high concentrations can limit percentage removal at a constant adsorbent dosage.^32^ In this investigation, MB solutions were prepared by diluting a 20 ppm stock solution and treated with 5 mg of SnS/SnO_*x*_ NPs and SnS/SnO_*x*_–GO–PVP NCs. The effect of the dye concentration was studied in the range of 4–10 for 100 min at 298 K. Furthermore, the effect of time was studied in 0–100 min intervals at 298 K, and the effect of pH on the adsorption of MB by the NPs and NC was also explored in the range of 4–9, with an adsorbent dose of 5 mg for a 4 ppm dye concentration solution, as these were the optimized parameters.

#### 2.6.2. Isotherm studies

Adsorption isotherm studies are used to determine how the equilibrium concentration of dye remaining in solution compares to the quantity of dye deposited on the adsorbent surface at a given temperature. These experiments examine the equilibrium distribution to determine if adsorption happens as a monolayer or multilayer, whether the surface is homogenous or heterogeneous, and how strongly dye molecules interact with the adsorbent. Fitting the data to isotherm models such as Langmuir, Freundlich, or Temkin models allows the determination of key parameters like the maximum adsorption capacity and adsorption intensity, which are essential for understanding the adsorption mechanism as well as for designing and optimizing dye removal systems in practice.^33^

The Freundlich adsorption isotherm is an empirical model that describes adsorption behavior on heterogeneous surfaces when binding sites have nonuniform energies. It allows for multilayer adsorption and is especially useful in systems where the adsorbent surface has structural or chemical imperfections. This model implies that the strongest binding sites are occupied first and that the adsorption intensity gradually diminishes as surface coverage increases. The linearized form of the Freundlich equation (eqn (4))^34^ is expressed as follows:4
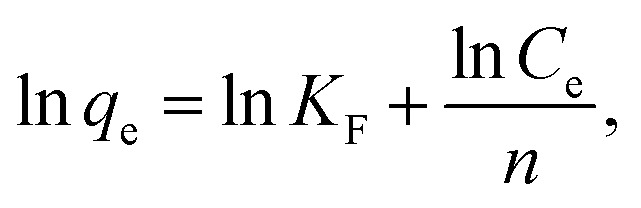
where *q*_e_ is the amount of dye adsorbed, 1/*n* and *K*_F_ are constants that are used to characterize the adsorption capacity and intensity, respectively, and *C*_e_ is the equilibrium concentration of dye remaining in the solution.

The Langmuir adsorption isotherm explains the process of adsorption on a homogeneous surface with similar and energetically equivalent binding sites. It is assumed that dye molecules form a homogeneous monolayer on the adsorbent surface and that each site may accommodate just one molecule,^35^ with no interactions between adsorbed species. This model is especially useful for systems where adsorption is dominated by specialized, localized interactions like electrostatic attraction or chemisorption. According to the Langmuir model, adsorption initially rises with the dye concentration but finally achieves saturation when all active sites are filled. The linearized form of the Langmuir equation (eqn (5)) is given as follows:5
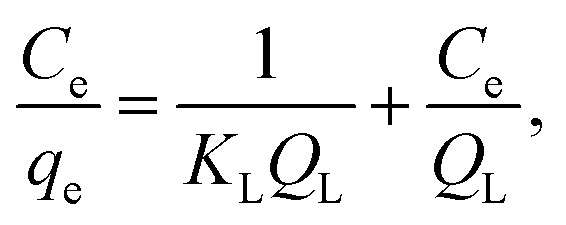
where *Q*_L_ is the maximum amount of dye adsorbed for the adsorbent surface, *K*_L_ is the Langmuir adsorption constant related to the affinity of binding sites.

The Temkin adsorption isotherm takes into consideration the effects of indirect interactions between adsorbate molecules and assumes that the heat of adsorption decreases linearly with increased surface coverage.^36^ Unlike the Langmuir model, which considers all sites to be energetically equivalent, the Temkin model acknowledges that adsorbent–adsorbate interactions lessen as more dye molecules occupy the surface. This makes the model ideal for systems in which adsorption causes a steady decrease in binding energy due to repulsive interactions or surface crowding. The Temkin equation also assumes a uniform distribution of binding energies up to a specific maximum value. The linearized form of the Temkin isotherm (eqn (6)) is expressed as follows:6
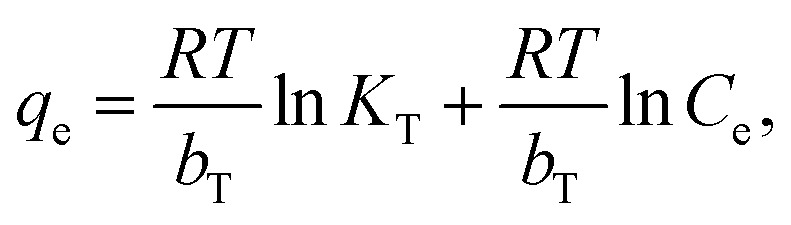
where *b*_T_ is a constant related to the heat of sorption associated with the parameter *b*_T_, *R* is the universal constant of gases, and *T* is the absolute temperature.

#### 2.6.3. Kinetic studies

The pseudo-second-order kinetic model is widely applied to describe adsorption processes where the rate-limiting step is controlled by chemisorption involving valence forces, electron sharing, or exchange between the adsorbate and the adsorbent surface. This model is capable of accurately representing adsorption behaviour over a wide concentration range and is often preferred because it provides reliable estimates of the equilibrium adsorption capacity. A good fit to this model indicates that the adsorption mechanism is strongly influenced by specific interactions rather than physical diffusion. For instance, phosphate on alunite^37^ have been successfully described by pseudo-second-order kinetics, with high correlation coefficients and physically meaningful *q*_e_ and *k*_2_ values. Extensions of second-order approaches have also been used to handle site heterogeneity and hysteretic adsorption–desorption of organic contaminants in soils.^38^ Moreover, comparisons of linear and nonlinear fitting methods for dye biosorption show that nonlinear regression applied to the pseudo-second-order model provides more reliable kinetic parameters and better represents the full time-dependent uptake profile.^39^ The pseudo-second-order model is expressed (eqn (7)) as follows:7
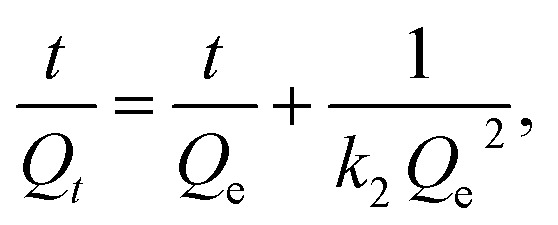
where *Q*_*t*_ is the amount of the adsorbate taken up by the adsorbent at time *t*, *Q*_e_ is the amount of the solute adsorbed at equilibrium, and *k*_2_ is the rate constant of adsorption.

### 2.7. Experimental procedure for the selective reduction of nitrobenzene to azobenzene

A mixture of nitrobenzene (1.0 mmol), sodium borohydride (2.0 equiv.), SnS/SnO_*x*_–GO–PVP NC (5 mg) and methanol (2.0 mL) was taken in a 50 mL round-bottom flask. The reaction mixture was refluxed for 2 hours in the presence of light. The formation of the product was monitored by TLC. Upon the completion of the reaction, the catalyst was separated using filter paper (Whatman no. 40). The filtrate was concentrated under vacuum, followed by the extraction of the residue with ethyl acetate and water. The organic part was washed with brine and dried over Na_2_SO_4_. After filtration, the filtrate was concentrated under reduced pressure. Column chromatography over silica gel (60–120 mesh) was performed using a mixture of ethyl acetate and petroleum ether (1 : 9) as an eluting solvent. A bright orange solid was obtained. The formation of pure azobenzene was confirmed by ^1^H and ^13^C NMR spectroscopic analyses.

### 2.8. Experimental procedure for the selective oxidation of benzoin

A mixture of benzoin (1.0 mmol) and SnS/SnO_*x*_–GO–PVP NC (10 mg) was prepared in 2 mL of toluene in a round-bottom flask under reflux conditions for 1 h while being constantly stirred with a magnetic stirrer. The progress of the reaction was monitored by TLC. The catalyst was then separated using filter paper (Whatman no. 40). After that, the filtrate was concentrated under vacuum, followed by the extraction of the residue with ethyl acetate and water. The organic part was washed with brine, dried over Na_2_SO_4_, and purified using short-column chromatography over silica gel (60–120 mesh) using a mixture of ethyl acetate and petroleum ether (1 : 9) as an eluting solvent to obtain a yellow solid. The melting point of this solid was determined to be 95 °C (reported mpt = 97 °C). Further, ^1^H NMR spectroscopic analysis confirmed the formation of pure benzil.

## 3. Results and discussion

### 3.1. FTIR spectroscopy

**Fig. 2:**
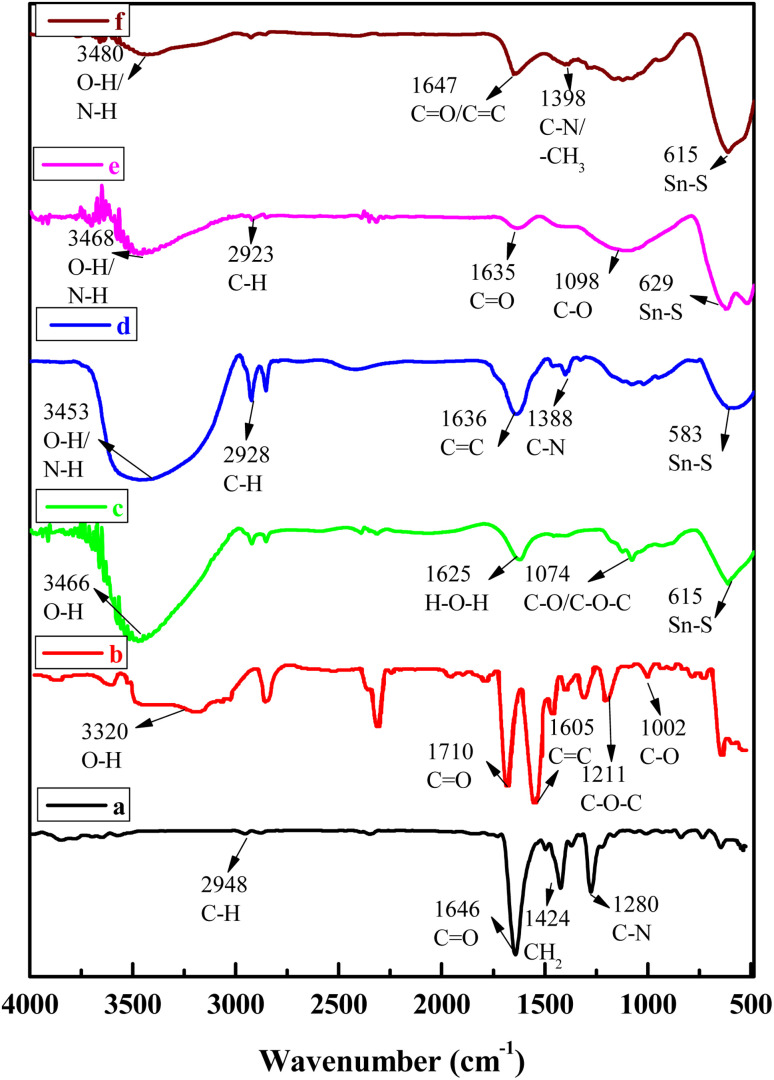
Comparative FTIR spectra of (a) PVP, (b) GO, (c) SnS/SnO_*x*_ NPs, (d) SnS/SnO_*x*_ NPs after adsorption, (e) SnS/SnO_*x*_–GO–PVP NC, and (f) SnS/SnO_*x*_–GO–PVP NC after adsorption.

The individual components' FTIR spectra showed the expected signatures, as shown in Fig. 2. PVP showed the C–H stretching at 2948 cm^−1^, pyrrolidone C=O at 1646 cm^−1^, CH_2_ bending at 1424 cm^−1^ and C–N at 1280 cm^−1^.^40^ GO showed a broad O–H band at 3320 cm^−1^, C=O at 1710 cm^−1^, graphitic C=C at 1605 cm^−1^, and oxygenated C–O/C–O–C bands at 1002–1211 cm^−1^.^41^ SnS/SnO_*x*_ NPs showed surface O–H/water bands at 3466 and 1625 cm^−1^, as well as the diagnostic Sn–S stretch at 615 cm^−1^.^42^ MB-specific bands appeared, such as those corresponding to C–H at 2928 cm^−1^, C–N at 1388 cm^−1^, and aromatic C=C/C=N at 1636 cm^−1^, after MB adsorption on SnS/SnO_*x*_ NPs; however, the peaks corresponding to Sn–S vibration persisted, showing that Sn–S and adsorbed MB interacted *via* surface H-bonding and electrostatic interactions. The strong hydrogen bonding and coordination between PVP's carbonyl groups, GO's oxygenated groups, and SnS's surface sites were consistent with the spectra of SnS/SnO_*x*_–GO–PVP NC, which showed a broad OH/NH feature at 3468 cm^−1^, retained C–H bands, and a downshifted/broadened carbonyl/C=C region at 1635 cm^−1^; the Sn–S band at 615–629 cm^−1^ verified that SnS was still present. After MB adsorption on NC, MB bands appeared, and the C–N/CH_3_ band at 1398 cm^−1^ reappeared along with a slight shift in the C=O/C=C region to 1647 cm^−1^ and the retention of the broad OH/NH band. This suggests a combined adsorption mechanism that includes hydrogen bonding with OH/NH/C=O groups, π–π stacking with GO graphitic domains, as shown by C=C/C=O perturbation, electrostatic attraction to oxygenated GO sites, and additional surface interactions with SnS.

### 3.2. XRD

**Fig. 3:**
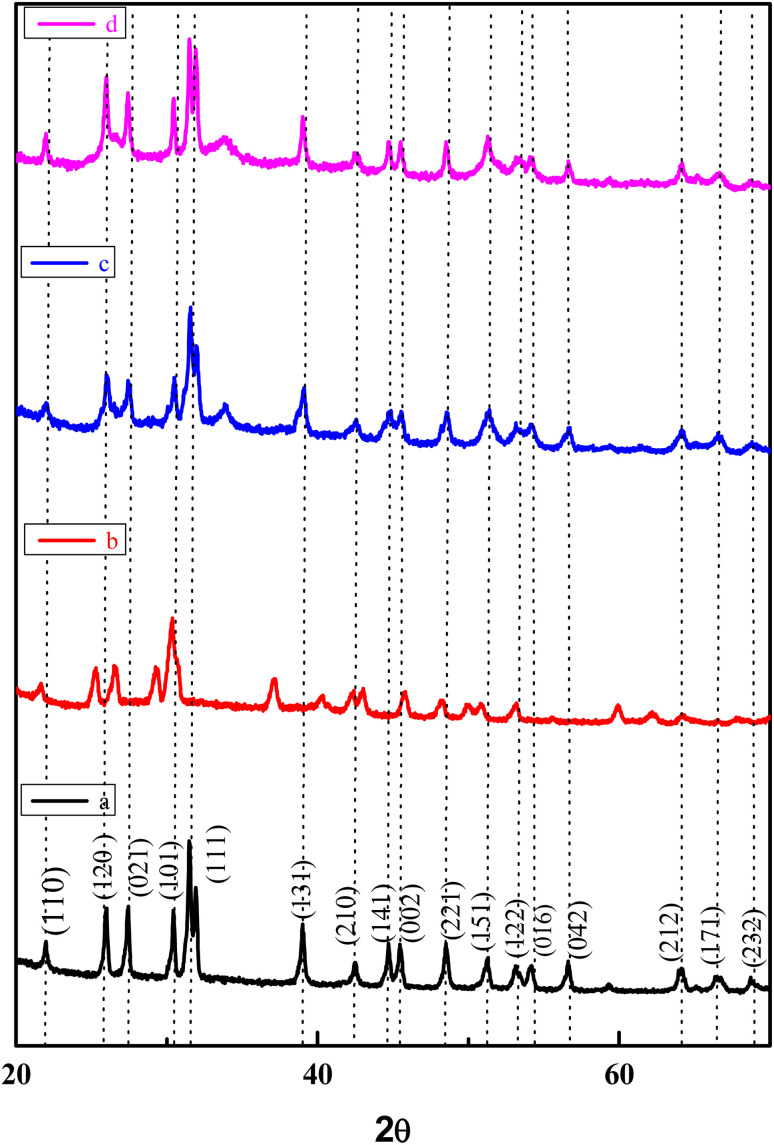
Comparative XRD spectra of (a) SnS/SnO_*x*_ NPs, (b) SnS/SnO_*x*_ NPs after adsorption, (c) SnS/SnO_*x*_–GO–PVP NC, and (d) SnS/SnO_*x*_–GO–PVP NC after adsorption.

The comparative XRD spectra of SnS/SnO_*x*_ NPs, SnS/SnO_*x*_ NPs after adsorption, SnS/SnO_*x*_–GO–PVP NC, and SnS/SnO_*x*_–GO–PVP NC after adsorption are shown in Fig. 3, and the crystallite size was calculated by the Scherrer formula (eqn (8)),^43^8
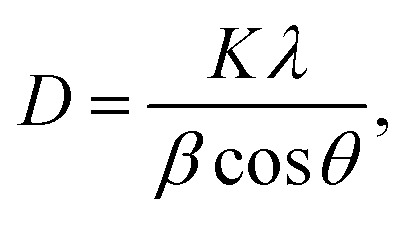
where, *D* = average crystallite size, *K* = Scherrer constant (0.94), *λ* = wavelength, *β* = full-width at half-maximum (FWHM), in radians, and *θ* = Bragg angle.

The XRD reflections of SnS/SnO_*x*_ NPs before adsorption appeared at 2*θ* values of 21.90°, 25.89°, 27.42°, 30.48°, 31.67°, 38.97°, 42.37°, 44.58°, 45.43°, 48.48°, 51.20°, 53.07°, 54.09°, 56.64°, 64.11°, 66.66°, and 68.70°, corresponding to the (110), (120), (021), (101), (111), (131), (210), (141), (002), (221), (151), (122), (016), (042), (212), (171), and (232)^44,45^ planes of SnS, respectively. By contrast, after MB adsorption, these peaks shifted slightly to 21.68°, 25.79°, 27.10°, 30.09°, 31.39°, 38.68°, 42.23°, 44.48°, 45.22°, 48.21°, 51.02°, 52.89°, 53.82°, 56.44°, 63.91°, 66.34° and 68.58°, respectively.^46^ These small downward shifts in several major reflections indicated a minor increase in spacing, caused by surface relaxation after dye attachment. Correspondingly, the calculated crystallite size exhibited only a marginal rise from 2.16 nm to 2.21 nm, which is an apparent increase arising from slight peak narrowing as the dye coating reduces surface microstrain. Before adsorption, SnS/SnO_*x*_–GO–PVP NC's characteristic peaks appeared at 21.94°, 26.02°, 27.38°, 30.44°, 31.63°, 33.83°, 38.93°, 42.50°, 44.71°, 45.56°, 48.45°, 51.16°, 53.20°, 54.05°, 56.77°, 65.07° and 66.62°, whereas after adsorption, they shifted to 21.94°, 25.85°, 27.38°, 30.44°, 31.63°, 33.83°, 38.93°, 42.50°, 44.71°, 45.39°, 48.45°, 51.16°, 53.20°, 54.05°, 56.60°, 64.07°, 66.62° and 68.83°, respectively.^47^ These peak shifts indicated a crystallite size reduction from 1.28 nm to 1.09 nm. This decrease does not represent the actual fragmentation of crystallites; instead, it reflects increased microstrain and structural disorder caused by dye interaction with the multicomponent SnS/SnO_*x*_–GO–PVP interface. After adsorption, all characteristic diffraction peaks of SnS and its nanocomposite remained at the same 2*θ* positions, confirming that the crystal structure is preserved. No additional peaks corresponding to the adsorbate were observed, indicating that adsorption occurs predominantly on the surface without altering the bulk crystallinity.

### 3.3. TGA

The thermal analysis of SnS/SnO_*x*_ NPs and SnS/SnO_*x*_–GO–PVP NC is shown in Fig. S1. SnS/SnO_*x*_ NPs show a distinct loss-gain-loss pattern in their thermal behavior. SnS/SnO_*x*_ NPs exhibit a weight loss of up to around 365 °C as a result of surface functional group breakdown, evaporation of adsorbed water, and the beginning of sulfur volatilization as SnS partly breaks down (SnS → Sn_*x*_S_*y*_ + S_3_). The most diagnostic characteristic is the unexpected weight gain between 365 °C and 492 °C.^47^ SnS oxidizes in the presence of trace oxygen, absorbing O atoms and generating heavier oxide phases. SnS + 2O_2_ → SnO_2_ + SO_2_↑ is the main reaction, which means that even if SO_2_ escapes, the solid absorbs oxygen mass. Sulfate intermediates follow the reaction SnS + 2O_2_ → SnSO_4_ as they develop. Above 492 °C, the weight drops due to the thermal decomposition of oxidized species, SnSO_4_ → SnO_2_ + SO_3_↑, releasing gaseous SO_3_ and pyrolyzing the remaining carbonaceous particles. In SnS/SnO_*x*_–GO–PVP NC, the initial loss up to 354 °C relates to water evaporation and oxygenated group breakdown, whereas that between 354 °C and 543 °C involves C–N/C=O bond breaking and the formation of a stable SnS–carbonaceous matrix, suggesting its higher thermal stability than that of SnS/SnO_*x*_ NPs.

### 3.4. SEM

**Fig. 4:**
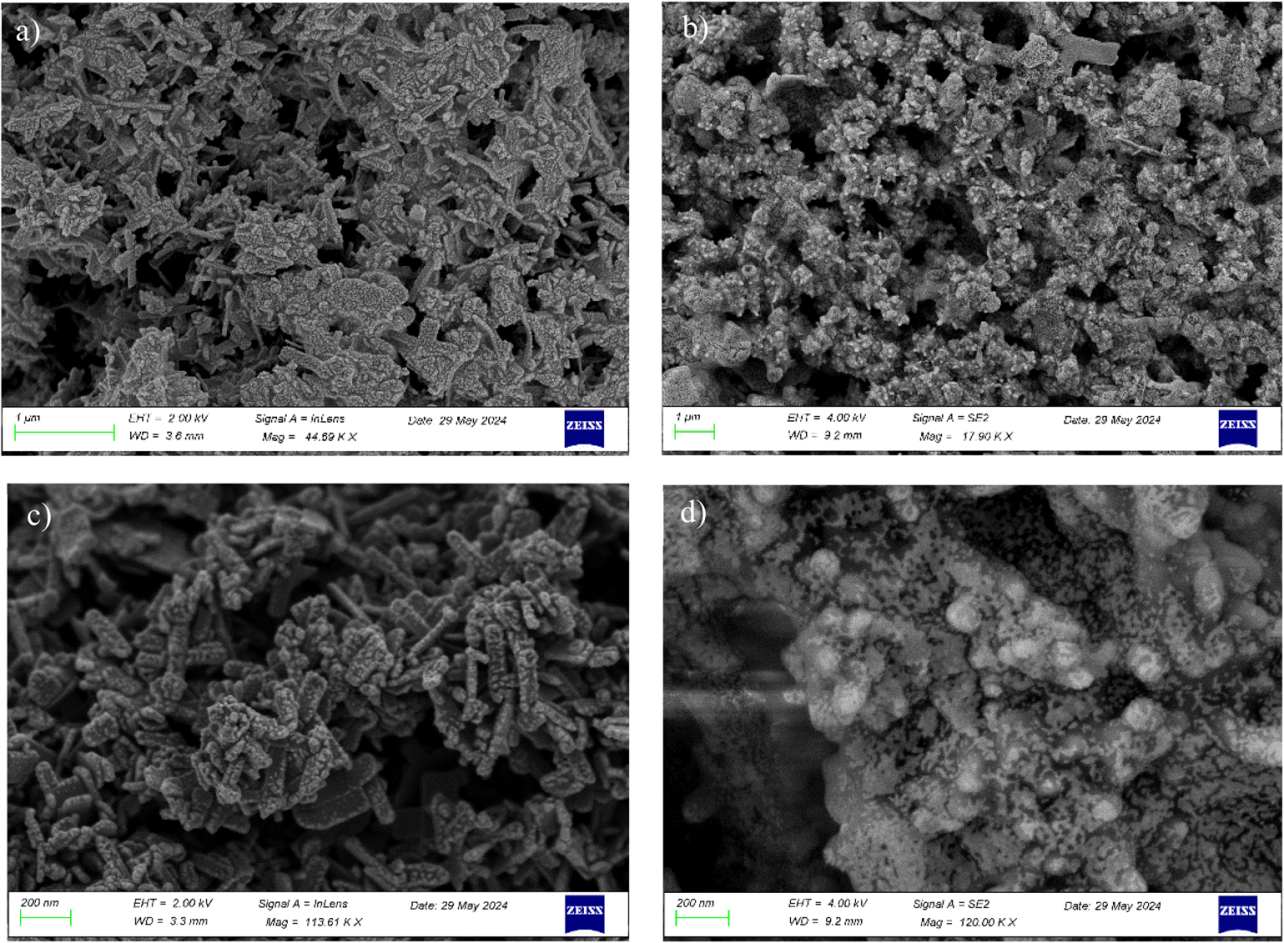
Scanning electron micrographs of (a) SnS/SnO_*x*_ NPs, (b) SnS/SnO_*x*_–GO–PVP NC, (c) SnS/SnO_*x*_ NPs after adsorption, and (d) SnS/SnO_*x*_–GO–PVP NC after adsorption.

The scanning electron micrographs of SnS/SnO_*x*_ NPs, SnS/SnO_*x*_–GO–PVP NC, SnS/SnO_*x*_ NPs after adsorption, and SnS/SnO_*x*_–GO–PVP NC after adsorption are shown in Fig. 4(a–d). The SEM image of SnS/SnO_*x*_ NPs reveals a porous, rod-/rectangular-like, flaky morphology caused by the anisotropic development of Sn–S layers and poor interparticle binding. These characteristics provide open spaces and uneven, heterogeneous surfaces, resulting in limited but accessible adsorption sites. In contrast, SnS/SnO_*x*_–GO–PVP NC has a hierarchical design, with tiny SnS nanorods scattered and perhaps partially embedded inside the larger GO/PVP matrix. This hybrid structure affords a much larger surface area, numerous functional groups, and interconnected porosity, providing the NC with a noticeably more adsorption-active surface than that of SnS/SnO_*x*_ NPs. SnS/SnO_*x*_ NPs exhibit evident pore filling, surface masking, and decreased roughness following MB adsorption, indicating MB surface coverage but only mild structural consolidation. SnS/SnO_*x*_–GO–PVP NC, however, experiences a far more noticeable change: the SnS rods are covered in a dye-rich matrix, the surface appears very packed and agglomerated, and the previously open interflake gaps are densely filled. Because MB interacts concurrently with SnS (by electrostatic binding), GO sheets (*via* π–π stacking), and PVP (*via* dipole-hydrogen bonding), it can offer multipoint anchoring, which SnS/SnO_*x*_ NPs cannot, leading to a greater densification. Due to the synergistic interactions and increased active site density, SnS/SnO_*x*_–GO–PVP NC exhibits a greater MB adsorption capacity, which is consistent with improved dye absorption in carbon–polymer–sulfide hybrid adsorbents.

### 3.5. BET

**Fig. 5:**
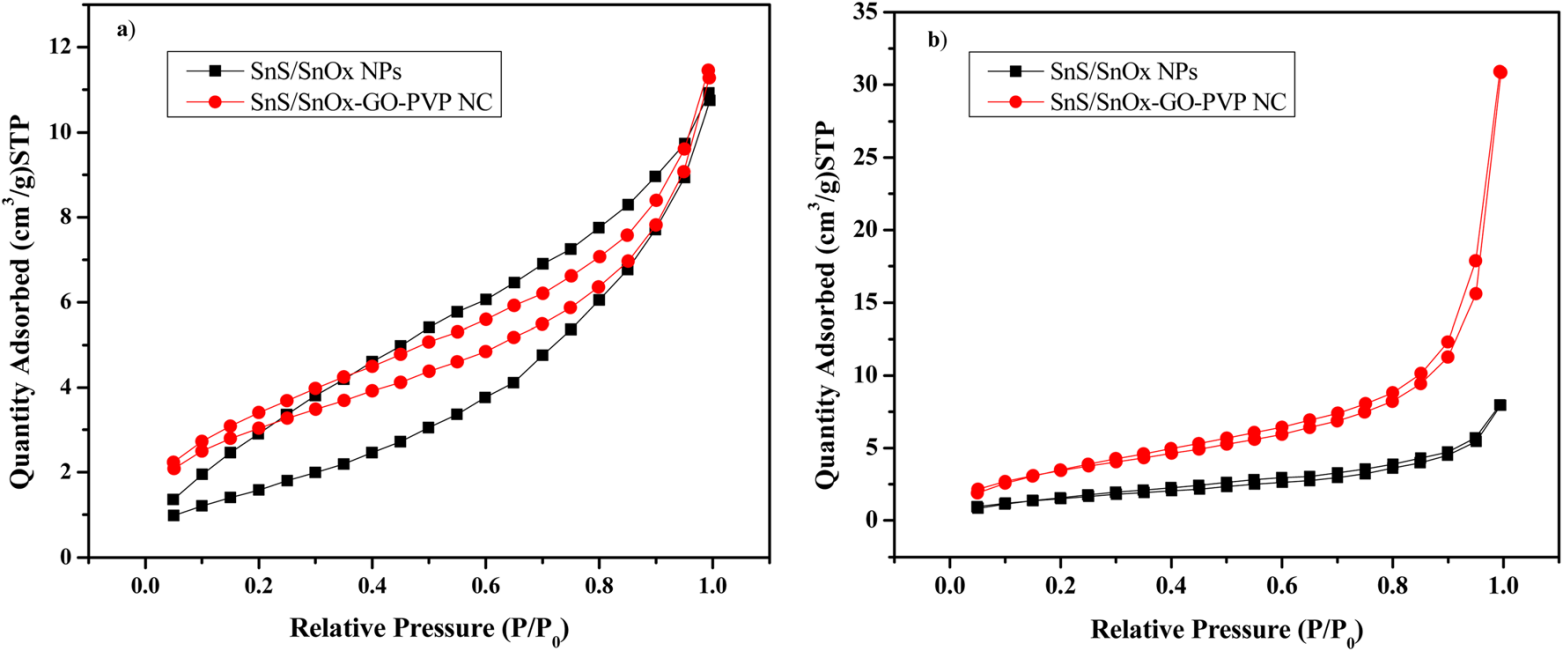
BET surface area analysis results: N_2_ adsorption–desorption isotherms of (a) SnS/SnO_*x*_ NPs and SnS/SnO_*x*_–GO–PVP NC before adsorption and (b) SnS/SnO_*x*_ NPs and SnS/SnO_*x*_–GO–PVP NC after adsorption.

The multipoint BET surface area analysis of SnS/SnO_*x*_ NPs and SnS/SnO_*x*_–GO–PVP NC before adsorption and SnS/SnO_*x*_ NPs and SnS/SnO_*x*_–GO–PVP NC after adsorption (Fig. 5) showed that SnS/SnO_*x*_ NPs possess a relatively low specific surface area of 6.39 m^2^ g^−1^ before MB uptake, which declines to 5.79 m^2^ g^−1^ after adsorption, indicating that the dye molecules occupy a noticeable fraction of the limited surface and may block pores. SnS/SnO_*x*_–GO–PVP NC has a higher surface area of 11.22 m^2^ g^−1^ before MB adsorption, which increases to 13.22 m^2^ g^−1^. The increase in the multipoint BET surface area may suggest that adsorption and subsequent sample treatment induce the structural rearrangement of the GO–PVP network, exposing previously inaccessible pores and surface sites. This result is consistent with other studies that found that adding PVP and graphene-derived supports significantly increases the surface area and adsorption performance of tin sulfide materials.^48^ The advantage of using high-surface-area carbonaceous scaffolds is further highlighted by the fact that graphene-based hybrids have been demonstrated to achieve BET values significantly higher than those of bare sulfides, with reported specific areas up to 1700 m^2^ g^−1^ for N and S codoped porous carbon spheres.^49^ As a consequence, compared to the small surface area of SnS/SnO_*x*_ NPs, SnS/SnO_*x*_–GO–PVP NC yields the best outcome for MB removal because of its higher, post-adsorption BET area (13.2 m^2^ g^−1^), which indicates a more efficient and accessible porous structure.

The BET data reveal a clear difference in how the two materials respond to the adsorption process. Before adsorption, SnS/SnO_*x*_ NPs have a higher surface area (6.626 m^2^ g^−1^) (BJH adsorption) than SnS/SnO_*x*_–GO–PVP NC (5.502 m^2^ g^−1^). After adsorption, SnS's surface area decreases to 3.246 m^2^ g^−1^, indicating dye-induced pore obstruction. In contrast, SnS/SnO_*x*_–GO–PVP NC shows a significant increase in surface area following adsorption, going from 5.502 m^2^ g^−1^ to 8.375 m^2^ g^−1^. This may suggest structural rearrangement and exposure of extra accessible sites during dye contact. Tables S1 and S2 compare all the additional BET experimental results, such as BJH pore volumes and pore radii from the adsorption and desorption branches, for SnS/SnO_*x*_ NPs and SnS/SnO_*x*_–GO–PVP NC before and after MB dye adsorption.

### 3.6. XPS

**Fig. 6:**
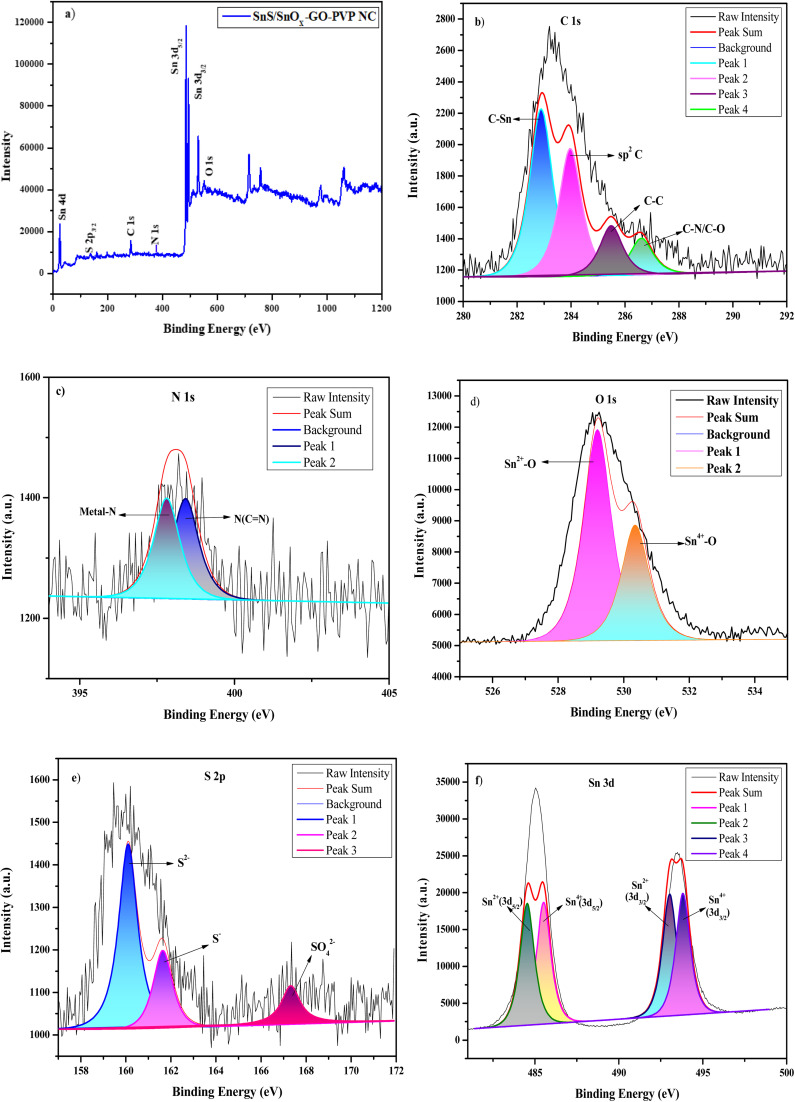
XPS spectra of SnS/SnO_*x*_–GO–PVP NC: (a) broad-scan spectrum and (b) C 1s, (c) N 1s, (d) O 1s, (e) S 2p, and (f) Sn 3d spectra.

The XPS broad-scan spectrum of SnS/SnO_*x*_–GO–PVP NC confirms the presence of the essential elements carbon (C), nitrogen (N), oxygen (O), sulphur (S), and tin (Sn), as shown in Fig. 6. The C 1s spectrum shows a peak at 282.88 eV, corresponding to C–Sn bonding, confirming the interfacial interaction between the carbon framework and tin species. The peaks at 283.96 eV and 285.48 eV are assigned to sp^2^ carbon and C–C/C–H bonds, respectively, while the component at 286.62 eV arises from the C–N/C–O functionalities of PVP and oxygenated GO.

The N 1s spectrum exhibits components at 397.80 eV (N–metal) and 398.40 eV (C=N), indicating the coordination of the polymer's nitrogen with tin species. Quantitative investigation of the O 1s spectra indicated that the oxygen species consisted of 51.32% lattice oxygen at 529.20 eV and 48.68% (Cal. S2) defect-related oxygen species at 530.35 eV. The majority of lattice oxygen validates the formation of the SnO_*x*_ framework, although the presence of a significant proportion of defect-related oxygen indicates the presence of many defective surface sites. These defect sites can serve as extra active centres for dye adsorption and catalytic reactions by increasing surface reactivity and promoting interactions with reactant molecules. As a result, the coexistence of lattice oxygen and defect-related oxygen species should help the SnS/SnO_*x*_–GO–PVP nanocomposite perform better in adsorption and catalysis.

The S 2p spectrum shows peaks at 160.07 eV and 161.63 eV, corresponding to sulfide species (S^2−^/S^−^), while the peak at 167.30 eV is assigned to sulfate (SO_4_^2−^), indicating partial surface oxidation. The Sn 3d spectrum reveals Sn^2+^ and Sn^4+^ states, with Sn 3d_5/2_ peaks at 484.57 eV and 485.51 eV, and the corresponding Sn 3d_3/2_ peaks at 493.02 eV and 493.79 eV, confirming the heterojunction formation of SnS and SnO_*x*_ in the nanocomposite.^50^

## 4. Adsorption study and redox catalytic activity

### 4.1. Methylene blue dye adsorption study

#### 4.1.1. Effect of concentration

**Fig. 7:**
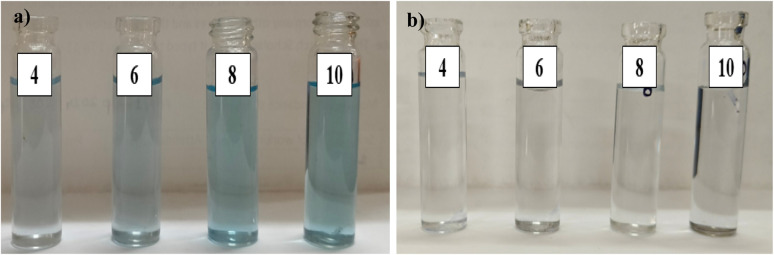
Methylene blue dye adsorption at concentrations of 4–10 ppm: (a) SnS/SnO_*x*_ NPs and (b) SnS/SnO_*x*_–GO–PVP NC.

**Fig. 8:**
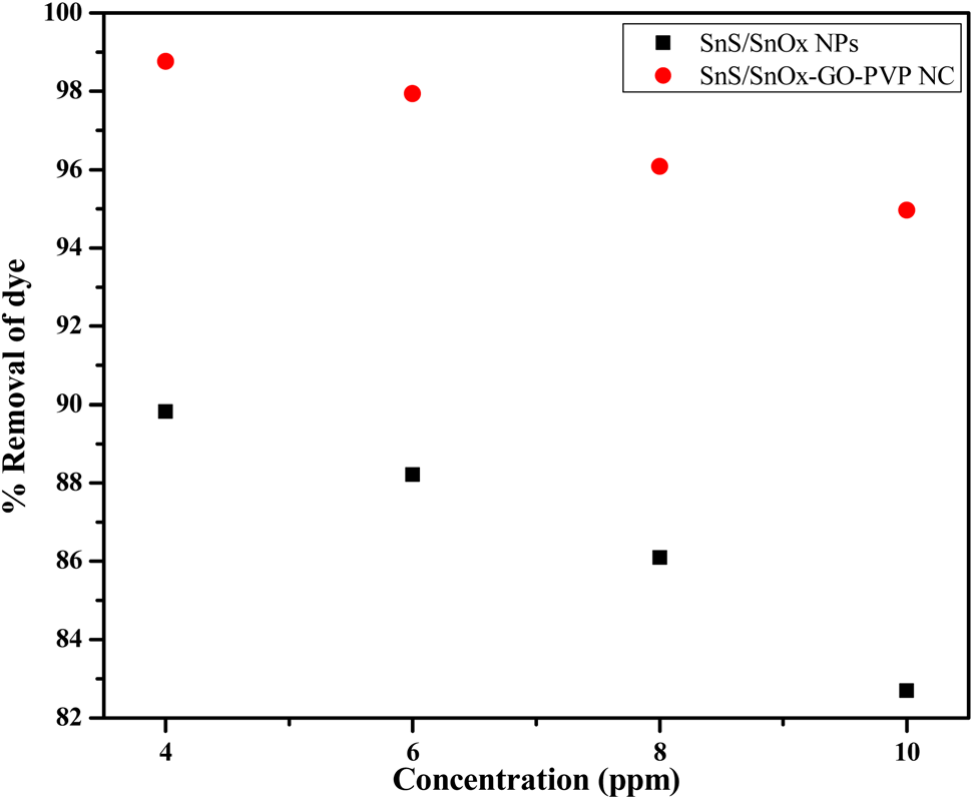
Effect of dye concentration on the adsorption of MB dye by SnS/SnO_*x*_ NPs and SnS/SnO_*x*_–GO–PVP NC.

Adsorption studies using 4–10 ppm MB dye demonstrate that SnS/SnO_*x*_–GO–PVP NC consistently outperforms the SnS/SnO_*x*_ NPs. Because a fixed adsorbent mass (5 mg in 10 mL) supplies a finite number of binding sites, the percent removal for both adsorbents decreases as the initial dye concentration increases; higher dye loads saturate the highest-energy sites, resulting in a larger percentage of dye in the solution. The GO–PVP matrix may be responsible for the NC's better performance and significantly lower removal efficiency decline, as PVP stabilizes and disperses SnS/SnO_*x*_ domains to avoid aggregation, and GO enhances the available surface area and provides oxygenated functional groups for electrostatic and π–π interactions with cationic MB.^51^ SnS/SnO_*x*_–GO–PVP NCs' increased ability to sustain high removal across increasing dye concentrations is explained by a higher effective site density, advantageous adsorption methods, and decreased diffusion limits. The effect of the dye concentration on the adsorption and % removal of MB dye by SnS/SnO_*x*_ NPs and SnS/SnO_*x*_–GO–PVP NC is shown in Fig. 7, 8 and Table S3.

#### 4.1.2. Effect of contact time

**Fig. 9:**
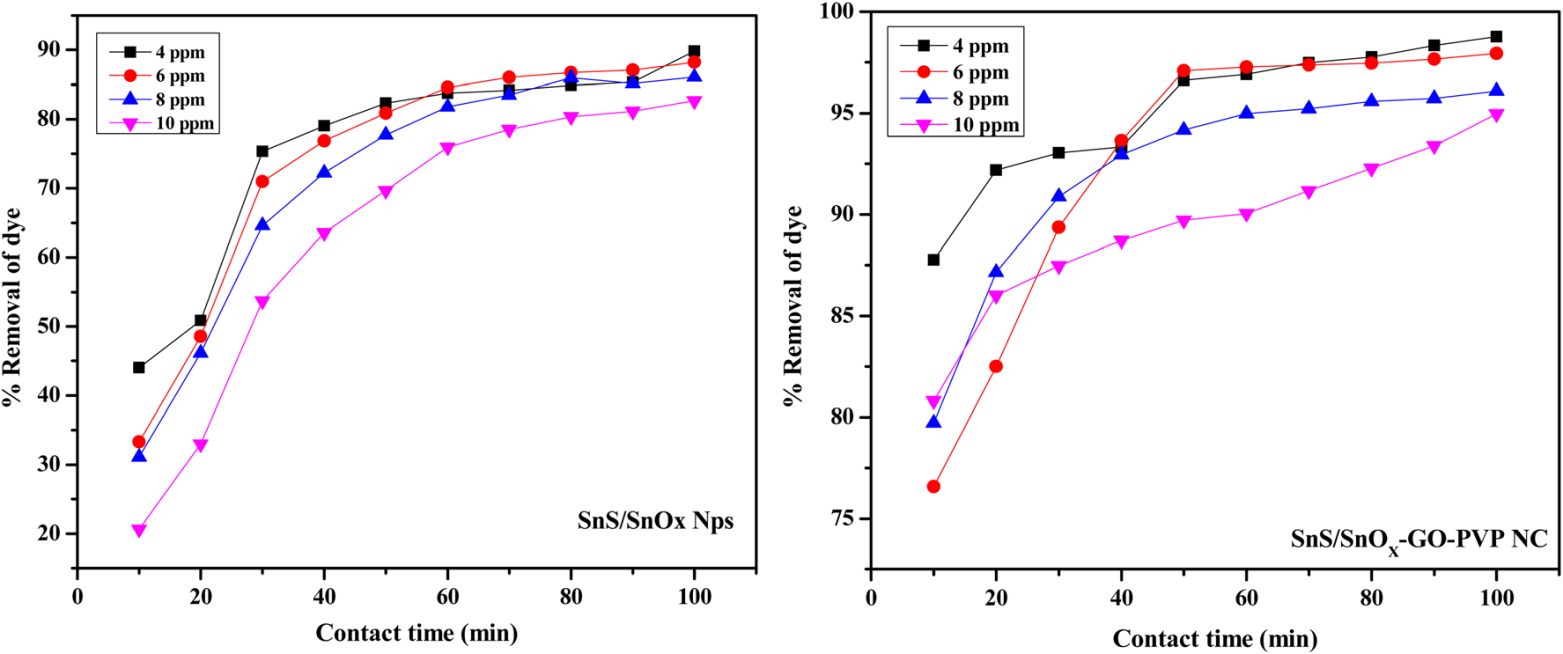
Effect of contact time on the removal of MB dye by SnS/SnO_*x*_ NPs and SnS/SnO_*x*_–GO–PVP NC.

The effect of contact time on MB removal clearly demonstrated that both SnS/SnO_*x*_ NPs and SnS/SnO_*x*_–GO–PVP NC approach equilibrium within 100 minutes, although their adsorption capabilities differ significantly (Fig. 9). At dye concentrations of 4–10 ppm, SnS/SnO_*x*_–GO–PVP NC exhibits significantly greater absorption (94.90–98.71%) than SnS/SnO_*x*_ NPs (82.56–89.66%). Lower contact times result in rapid dye removal up to 60 minutes, indicating that both SnS/SnO_*x*_ NPs and SnS/SnO_*x*_–GO–PVP NC have a large number of readily accessible active sites, allowing for fast initial adsorption before the process gradually slows as the surfaces approach saturation. Competition for limited binding sites is shown (Table S4) by the steady decrease in % removal for both materials with increasing MB concentration; however, the NC's slower fall validates its greater site density, improved dispersion, and stronger dye–surface interactions. SnS/SnO_*x*_–GO–PVP NC continuously outperforms SnS/SnO_*x*_ NPs because of its improved structural and chemical functionality, and 100 minutes are enough for near-complete adsorption.

#### 4.1.3. Effect of the pH of the solution

**Fig. 10:**
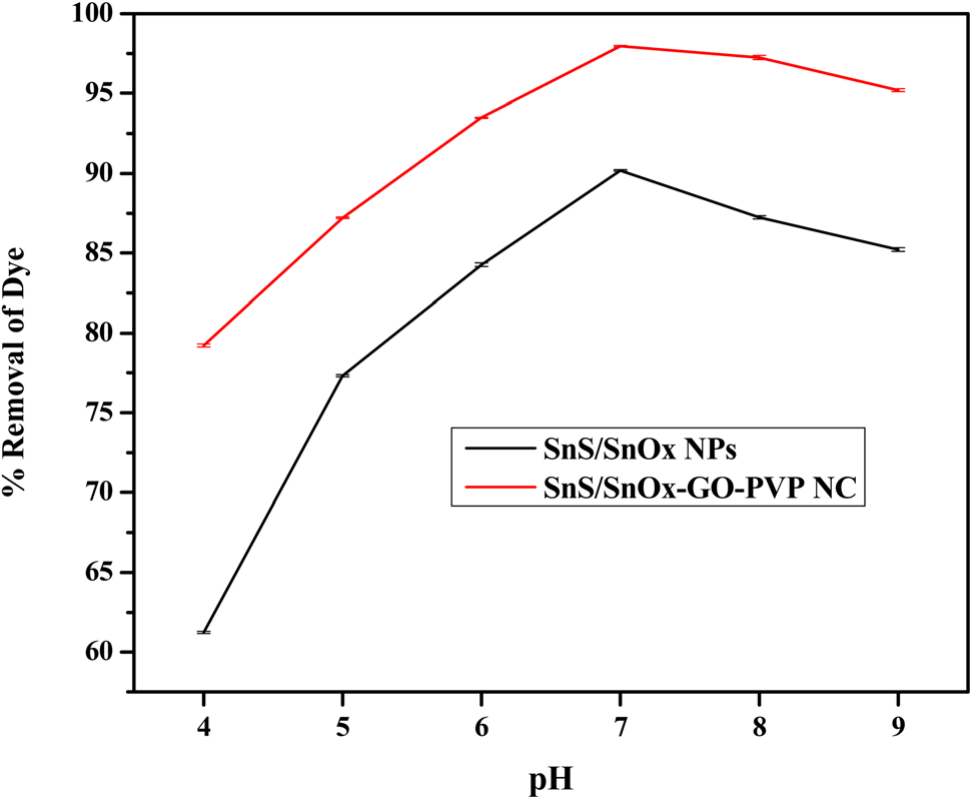
Effect of pH on the removal of MB dye by SnS/SnO_*x*_ NPs and SnS/SnO_*x*_–GO–PVP NC.

The effect of pH on the adsorption of MB dye by SnS/SnO_*x*_ NPs and SnS/SnO_*x*_–GO–PVP NC was investigated in the pH range of 4–9 (Fig. 10). The adsorption efficiency increases with increasing pH and reaches the maximum at pH 7. At pH 7, SnS/SnO_*x*_–GO–PVP NC removes the dye with a substantially higher effectiveness (98.56%) than SnS/SnO_*x*_ NPs (89.80%). At lower pH levels, extra H^+^ ions compete with positively charged MB molecules for accessible adsorption sites, resulting in decreased adsorption. As the pH rises, the surface of the adsorbent becomes more negatively charged, increasing the electrostatic interaction between the adsorbent and the cationic MB dye molecules. The NC's higher adsorption efficiency may be due to the synergistic impact of SnS, GO, and PVP, which afford a larger surface area, more active sites, better dispersion, and improved interactions with dye molecules. Beyond pH 7, the adsorption efficiency remains nearly constant, indicating that the adsorption process has approached saturation and that pH 7 is the optimum condition for MB dye removal.

#### 4.1.4. Adsorption isotherms

**Fig. 11:**
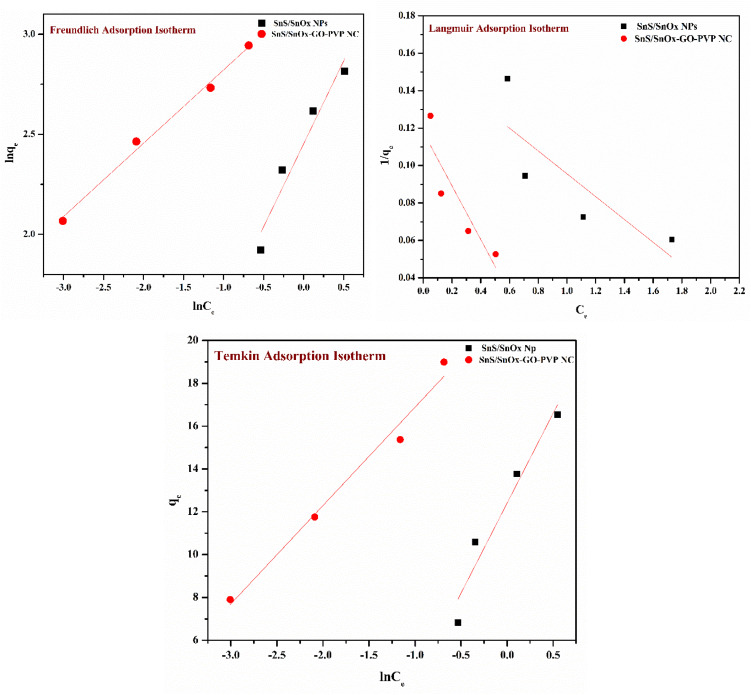
Linear plots of the Freundlich adsorption isotherm, Langmuir adsorption isotherm, and Temkin adsorption isotherm for the removal of MB dye using SnS/SnO_*x*_ NPs and SnS/SnO_*x*_–GO–PVP NC.

The design and optimisation of an adsorption system for removing dyes from aqueous solutions rely heavily on the adsorption isotherm. Thus, identifying the best correlation for the equilibrium curve is essential. Multiple adsorption isotherms can be used for studying the interaction between adsorbents and dyes.^52^ In this study, the Freundlich, Langmuir, and Temkin isotherm models were utilised to analyse the interface of the MB dye with SnS/SnO_*x*_ NPs and SnS/SnO_*x*_–GO–PVP NC. Fig. 11 shows the linear plots of all the three adsorption isotherms, and Table 1 gives the values of the adsorption parameters. Among the investigated models, the highest obtained *R*^2^ value is 0.92 for SnS NPs and 0.98 for SnS/SnO_*x*_–GO–PVP NC, indicating that the Freundlich isotherm offers the best fit for both adsorbents. For both SnS/SnO_*x*_ NPs (*n* = 1.21) and NC (*n* = 2.77), the Freundlich intensity parameter (*n*) is more than 1, indicating positive adsorption. Further evidence that SnS/SnO_*x*_–GO–PVP NC has a greater adsorption capacity and a better surface affinity for the dye molecules is provided by the larger *K*_F_ value of SnS/SnO_*x*_–GO–PVP NC (24.07 L mg^−1^) than that of SnS NPs (11.58 L mg^−1^). The result from Table 1 demonstrates that SnS/SnO_*x*_–GO–PVP NC exhibits superior adsorption affinity and a heterogeneous multilayer adsorption behaviour, as evidenced by the highest *R*^2^ values for the Freundlich and Temkin models and the higher Freundlich constants.

**Table 1:** Adsorption isotherm parameters for the adsorption of MB dye on SnS/SnO_*x*_ NPs and SnS/SnO_*x*_–GO–PVP NC

Isotherm type	Isotherm parameters	Values of isotherm parameters	
		SnS/SnO_*x*_ NPs	SnS/SnO_*x*_–GO–PVP NC
Freundlich isotherm	*n*	1.21	2.77
	*K*_F_ (L mg^−1^)	11.58	24.07
	*R* ^2^	0.92	0.98
Langmuir isotherm	*K*_L_	0.41	1.17
	*q*_m_ (mg g^−1^)	16.39	7.14
	*R* ^2^	0.52	0.72
Temkin isotherm	*b*_T_	305.21	557.8
	*K*_T_	4.38	4.67
	*R* ^2^	0.91	0.97

#### 4.1.5. Adsorption kinetics

**Fig. 12:**
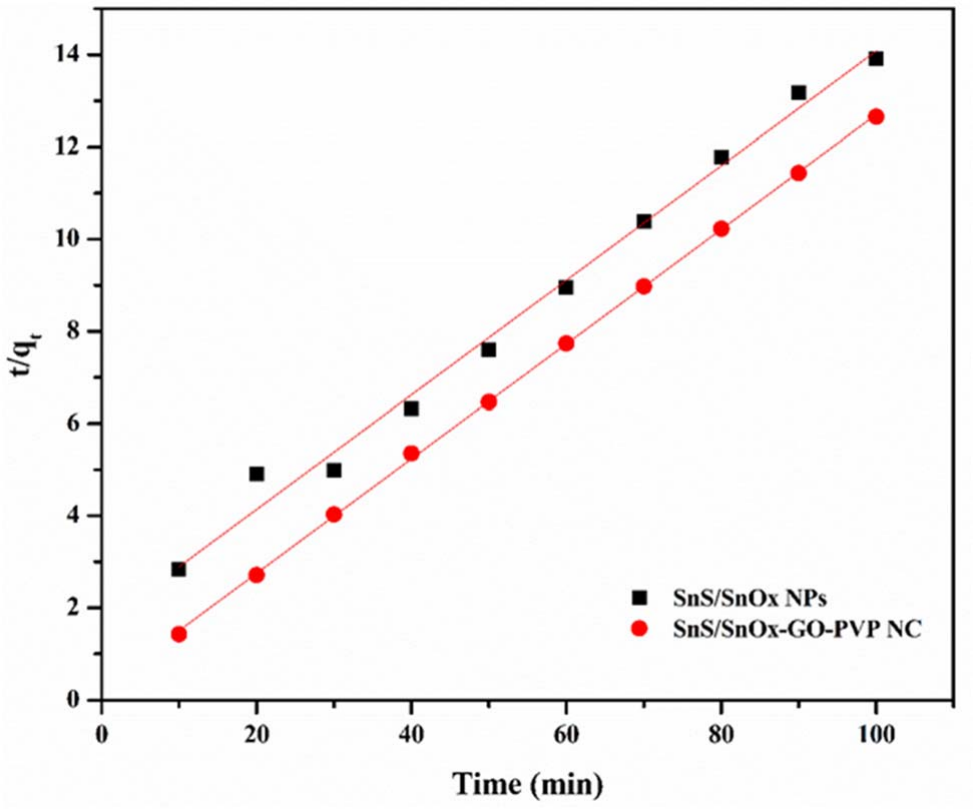
Linear plot of pseudo-second-order kinetics.

The linear plot for the pseudo-second-order model is shown in Fig. 12, with the corresponding parameters summarised in Table 2, The pseudo-second-order model demonstrates an excellent fit to the experimental data for both SnS/SnO_*x*_ NPs and SnS/SnO_*x*_–GO–PVP NC, with a very high correlation coefficient (*R*^2^) of 0.99 for both. The equilibrium adsorption capacity (*q*_e_) calculated from this model is 8.33 mg g^−1^ for SnS/SnO_*x*_ NPs and SnS/SnO_*x*_–GO–PVP NC, indicating that it improves the adsorption efficiency of the material, likely by enhancing the surface area, porosity, or functional group availability. The rate constant (*k*_2_) value for SnS/SnO_*x*_ NPs is 0.008 g mg^−1^ min^−1^, and for the SnS/SnO_*x*_–GO–PVP NC, it is 0.057 g mg^−1^ min^−1^.

**Table 2:** Adsorption kinetic parameters for the adsorption of MB dye on SnS/SnO_*x*_ NPs and SnS/SnO_*x*_–GO–PVP NC

Kinetic type	Parameters			
	Materials	*Q*_e_	*k*_2_	*R* ^2^
Pseudo-second-order	SnS/SnO_*x*_ NPs	8.33	0.008	0.99
	SnS/SnO_*x*_–GO–PVP NC	8.33	0.057	0.99

#### 4.1.6. Reusability test for dye adsorption

**Fig. 13:**
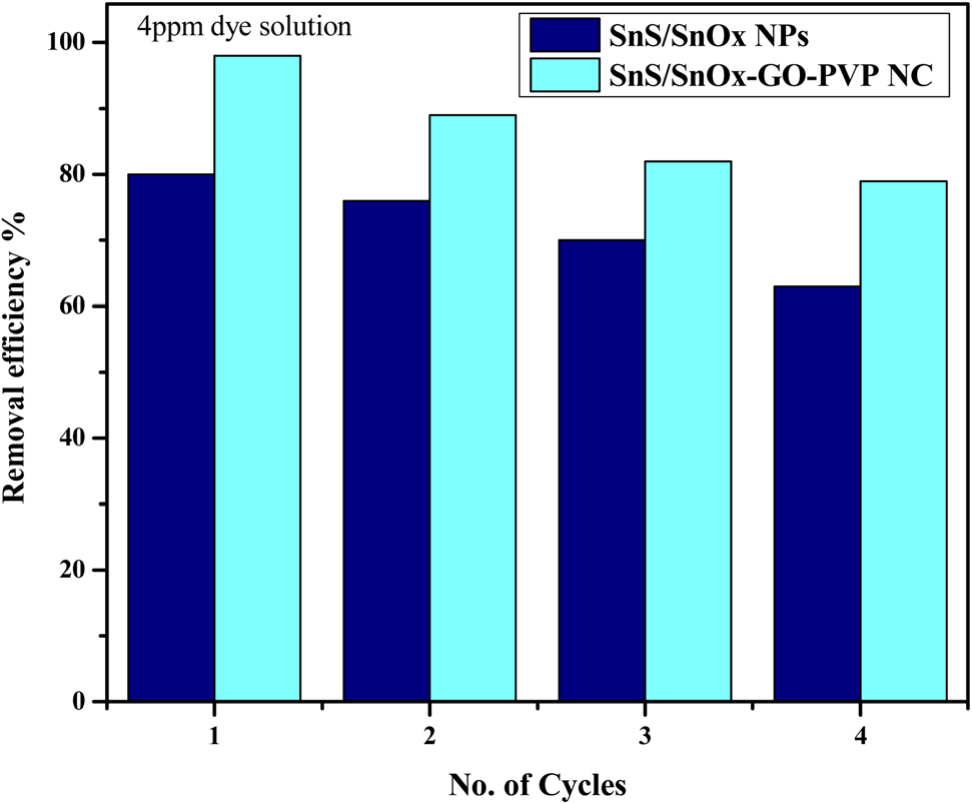
Reusability graph of SnS/SnO_*x*_ NPs and SnS/SnO_*x*_–GO–PVP NC for methylene blue dye at 4 ppm concentration.

After concluding the adsorption experiment, SnS/SnO_*x*_ NPs and SnS/SnO_*x*_–GO–PVP NC loaded with the MB dye were retrieved and subjected to multiple cycles of sonication and centrifugation to ensure the thorough removal of the dye. The dried samples were then reused for further dye adsorption to assess their recyclability. The efficiency of SnS/SnO_*x*_ NPs gradually decreased from 80% in the first cycle to 63% in the fourth cycle. In contrast, SnS/SnO_*x*_–GO–PVP NC exhibited significantly improved stability, maintaining catalytic efficiencies of 98%, 89%, 82%, and 79% in four successive cycles. In these cycles, we observed that the reusability of SnS/SnO_*x*_–GO–PVP NC is better than that of SnS/SnO_*x*_ NPs, as shown in Fig. 13.

### 4.2. Redox catalytic activity of SnS/SnO_*x*_–GO–PVP NC

#### 4.2.1. Study of the catalytic activity for the selective reduction of nitrobenzene to azobenzene

Nitrobenzene is a hazardous and persistent pollutant that is frequently released by the explosive and pesticide industries.^53,54^ The reduction of nitrobenzene to aniline has been extensively investigated. However, the selective synthesis of azobenzene from the abundantly available nitrobenzene remains considerably more demanding.^55^ Azobenzene and its derivatives have been excessively used as dyes, pH indicators, radical initiators, drugs, and biologically active compounds.^56^ Therefore, we selected the SnS/SnO_*x*_–GO–PVP NC to examine its catalytic activity in organic reactions because it outperformed SnS/SnO_*x*_ NPs in MB dye adsorption. The NC facilitates the efficient and highly selective transformation of nitrobenzene to azobenzene, without the formation of hazardous intermediates. The mixed Sn(ii)/Sn(iv) sites, conductive GO structure, and stabilizing PVP work together to afford great operational stability, fast electron transfer, and high catalytic efficiency, which makes it considerably more useful than traditional catalysts.

We initiated our investigations using the reduction of nitrobenzene as the model reaction. Initially, a mixture of nitrobenzene, methanol, and SnS/SnO_*x*_–GO–PVP was stirred at room temperature under visible light for 24 h. No detectable product formation was observed (entry 1, Table 3). Further, NaBH_4_ (1 equiv.) was added as the reducing reagent, which resulted in the formation of a trace amount of the product (entry 2, Table 3). When the same reaction mixture was used under reflux conditions, the yield of azobenzene significantly improved to 83% within 2 h (entry 3, Table 3). A controlled experiment was performed under dark conditions to study the role of visible light. Notably, no product formation could be seen, indicating that the reaction is not feasible in the absence of light (entry 4, Table 3). Next, reducing the catalyst loading to 2 mg resulted in a decrease in product yield to 60% (entry 5, Table 3). The effect of the solvent was examined using toluene and acetonitrile, which resulted in comparatively lower yields of 48% and 60%, respectively (entries 6 and 7, Table 3). Additionally, no conversion was observed in the absence of a catalyst (entry 8, Table 3), signifying that the reducing agent alone cannot mediate the reduction under similar conditions. For comparison, the catalytic activity of SnS NPs was studied under the same reaction conditions. The reaction yield was slightly lower (80%) but did not show a significant variation (entry 9, Table 3). After obtaining the optimized reaction conditions, the scope of nitrobenzenes was evaluated. Both electron-donating and electron-withdrawing groups were well-tolerated (Table 4). Furthermore, to shed light on the plausible mechanism of this conversion, a controlled experiment was carried out in the presence of a radical scavenger. The addition of 2 equivalents of 2,2,6,6-tetramethylpiperidinyloxy (TEMPO) in the reaction mixture containing nitrobenzene (1 mmol), NaBH_4_, and SnS/SnO_*x*_–GO–PVP NC under the optimized conditions suppressed the reaction yield of the desired product to ∼30%. The suppression of the reaction yield in the presence of the radical scavenger (TEMPO) suggests the involvement of radical intermediates during the catalytic process. These observations imply that the catalyst is likely to promote the activation of NaBH_4_*via* a single-electron transfer pathway, leading to the generation of reactive radicals that reduce nitrobenzene.

**Table 3:** Optimization of the reaction conditions for the reduction of nitrobenzene to azobenzene^a^

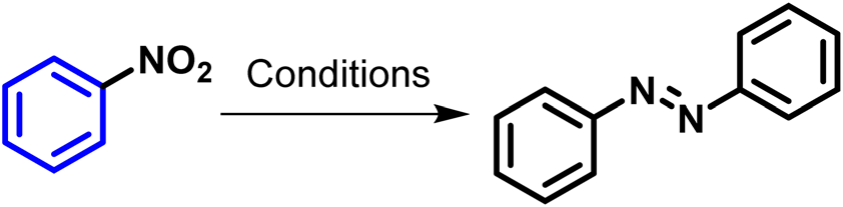						
Entry	Catalyst (amount)	Reducing agent	Solvent	Temperature	Time (h)	Conversion (%)
1	SnS/SnO_*x*_–GO–PVP (5 mg)	—	Methanol	R.T./visible light	24	N.R.
2	SnS/SnO_*x*_–GO–PVP (5 mg)	NaBH_4_ (1 equiv.)	Methanol	R.T./visible light	24	20
3	SnS/SnO_*x*_–GO–PVP (5 mg)	NaBH_4_ (2 equiv.)	Methanol	Reflux/visible light	2	83
4	SnS/SnO_*x*_–GO–PVP (5 mg)	NaBH_4_ (2 equiv.)	Methanol	R.T./dark	24	N.R.
5	SnS/SnO_*x*_–GO–PVP (2 mg)	NaBH_4_ (2 equiv.)	Methanol	Reflux/visible light	2	60
6	SnS/SnO_*x*_–GO–PVP	NaBH_4_ (2 equiv.)	Toluene	Reflux/visible light	2	48
7	SnS/SnO_*x*_–GO–PVP	NaBH_4_ (2 equiv.)	Acetonitrile	Reflux/visible light	4	60
8	SnS/SnO_*x*_–GO–PVP (5 mg)	NaBH_4_ (2 equiv.)	Methanol	Reflux/visible light	4	N.R.
9	SnS/SnO_*x*_ NPs (5 mg)	NaBH_4_ (2 equiv.)	Acetonitrile	Reflux/visible light	2	80

a Reaction conditions: nitrobenzene (1.0 mmol), SnS/SnO_*x*_–GO–PVP NC (5 mg), solvent (2 mL) (N.R. = no reaction; R.T. = room temperature).

**Table 4:** Substrate scope for the reduction of nitrobenzene to azobenzene^a^

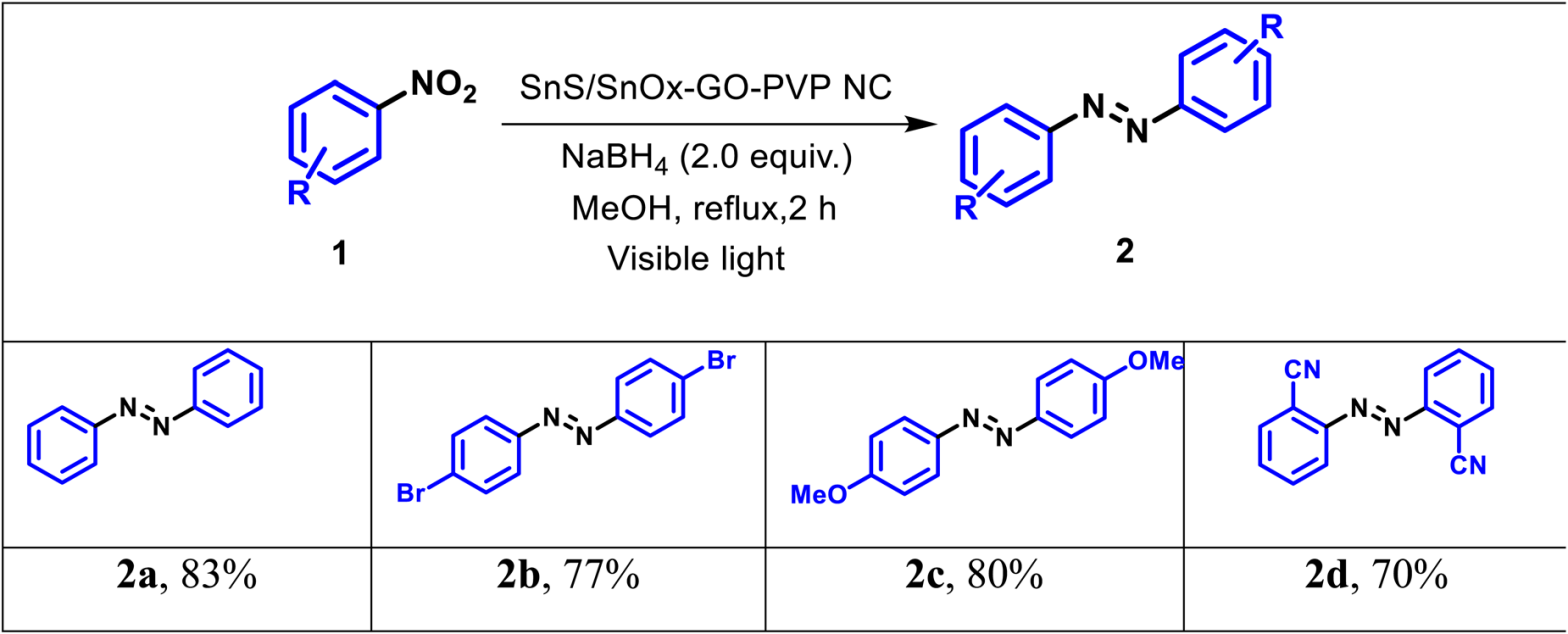

a Reaction conditions: 1 (1 mmol), 2 (1 mmol), NaBH_4_ (2.0 equiv.), and SnS/SnO_*x*_–GO–PVP NC (5 mg) in MeOH (2.0 mL) under reflux conditions in natural light for 2 h. The yields refer to pure and isolated products.

#### 4.2.2. Study of catalytic activity for the oxidation of alcohols

The catalytic efficacy of SnS/SnO_*x*_–GO–PVP NC was further assessed for the oxidation of alcohols. The selective oxidation of alcohols to their corresponding carbonyl compounds^57^ is a high-value transformation because these products serve as critical intermediates in fine chemical, agrochemical, perfume, and, most importantly, pharmaceutical syntheses. Liquid-phase catalytic oxidation is especially essential because it creates cleaner reaction pathways, prevents over-oxidation, and allows for greater control over selectivity.^58^ Benzoin oxidation to benzil is a well-studied process due to its α-dicarbonyl structure, which is essential for creating heterocycles, photoactive chemicals, and radical polymerization precursors. Benzil is commonly used as a photosensitizer, a synthetic building block, and a starting point for producing physiologically active compounds and chiral ligands. Using SnS/SnO_*x*_–GO–PVP NC in this reaction makes sense because the mixed Sn(ii)/Sn(iv) redox centres, oxygen-rich SnO_*x*_ domains, and high-surface-area GO help to accelerate electron transfer and stabilize key intermediates, allowing for the faster and more selective conversion of benzoin to benzil. In other words, the catalyst's efficacy is based on true structural synergy—not merely the presence of SnS—and the combined effects of the SnS/SnO_*x*_ heterojunction, GO dispersion, and PVP surface stability, all of which improve active-site accessibility and prevent catalytic deactivation.

We began the investigation using the oxidation of benzyl alcohol as the model reaction. A mixture of benzyl alcohol and SnS/SnO_*x*_–GO–PVP NC in toluene was stirred at ambient temperature for 1 hour. No detectable conversion was seen (entry 1, Table 5). Increasing the temperature of the reaction mixture to 100 °C led to 50% conversion after 1 hour (entry 2, Table 5). Subsequently, TBHP was introduced as the oxidising agent, which increased the yield to 60% within 30 min (entry 3, Table 5). When the reaction time was extended to 1 hour, the yield of benzaldehyde further increased to 85% (entry 4, Table 5). Following this, the solvents were varied. Under the same reaction conditions, dioxane and DCM afforded lower yields of 57% and 10%, respectively. Next, by reducing the catalyst amount to 5 mg, the yield decreased to 55% (entry 7, Table 5). Based on the optimized reaction conditions, the substrate scope was explored for a wider variety of alcohols, and both electron-donating and electron-withdrawing groups were well-tolerated (Table 6).

**Table 5:** Optimization of the reaction conditions for the oxidation of benzoin

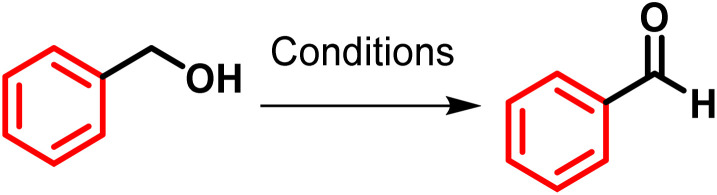					
Entry	Solvent	Oxidant	Temperature (°C)	Time (h)	Conversion^a^ (%)
1	Toluene	None	25	1.0	N.R.
2	Toluene	None	100	1.0	50
3	Toluene	TBHP	100	0.5	60
4	Toluene	TBHP	100	1.0	85
5	Dioxane	TBHP	100	1.5	57
6	DCM	TBHP	100	1.0	10
7	Toluene	TBHP	100	1.0	55^b^

a Reaction conditions: benzyl alcohol (1.0 mmol), SnS/SnO_*x*_–GO–PVP NC (10 mg) in 2 mL of the solvent.

b 5 mg of SnS/SnO_*x*_–GO–PVP NC.

**Table 6:** Substrate scope for the oxidation of alcohols^a^

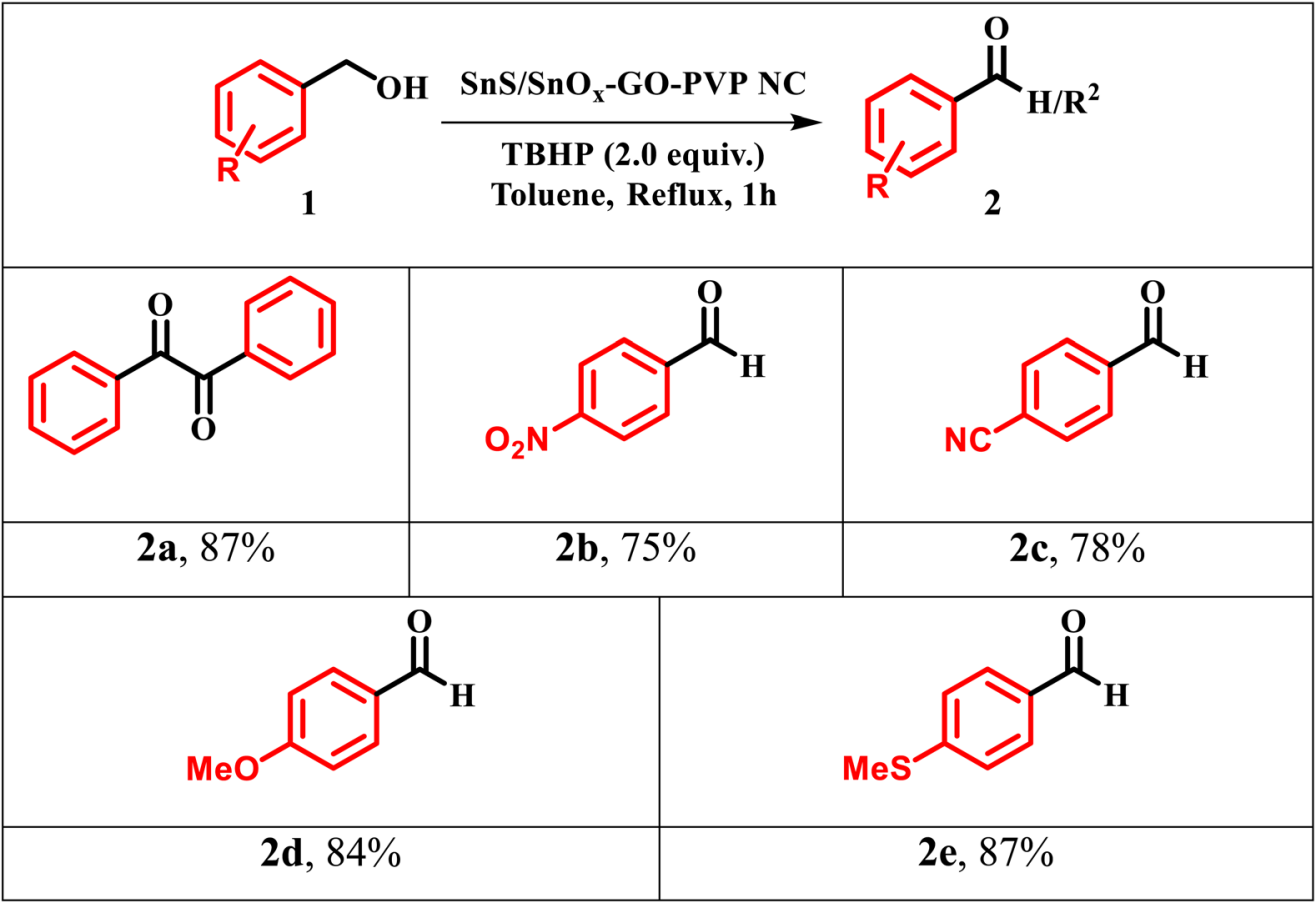

a Reaction conditions: alcohol (1.0 mmol), SnS/SnO_*x*_–GO–PVP NC (10 mg), TBHP (2.0 equiv.), toluene (2 mL) under reflux conditions. The yields refer to pure and isolated products.

#### 4.2.3. Recyclability test

**Fig. 14:**
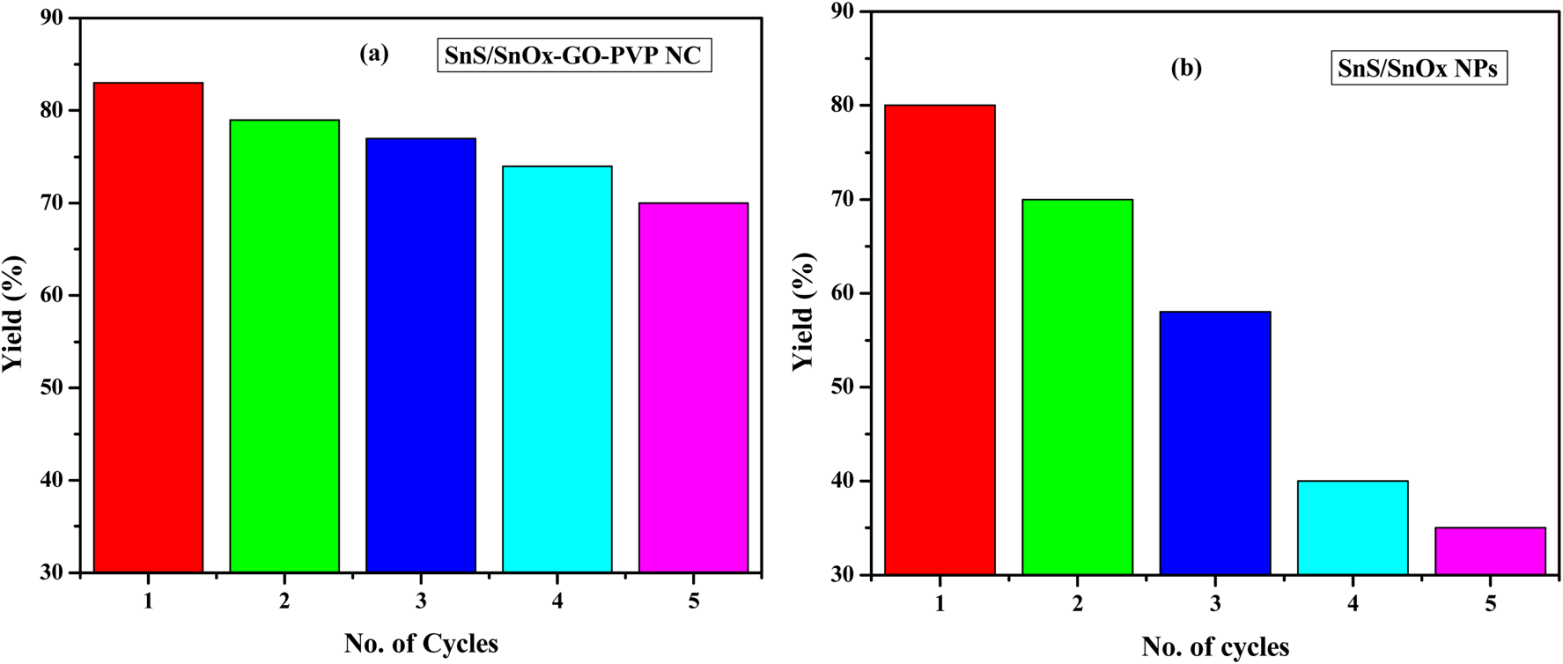
Recyclability studies of (a) SnS/SnO_*x*_–GO–PVP NC and (b) SnS/SnO_*x*_ NPs.

The recyclability of SnS/SnO_*x*_–GO–PVP NC and SnS NPs was assessed during the catalytic conversion of nitrobenzene to azobenzene. After each reaction cycle, the catalysts were filtered and carefully washed with methanol. Results show that SnS/SnO_*x*_–GO–PVP NC may be reused up to five times without a significant variation in the catalytic activity. However, a modest but persistent drop in the azobenzene yield is observed, which might be attributed to little catalyst loss during the recycling process. However, SnS/SnO_*x*_ NPs show more suppression in the yield of azobenzene, indicating lower structural stability compared to SnS/SnO_*x*_–GO–PVP NC. The recyclability data in Fig. 14 reveal that SnS/SnO_*x*_–GO–PVP NC preserves its catalytic activity comparatively better than SnS NPs. This implies that the catalyst structure is mostly intact during the reaction cycles. In contrast, SnS NPs rapidly lose activity, exhibiting yields of 80% in the first cycle, 70% in the second cycle, 58% in the third cycle, 40% in the fourth cycle, and just 35% in the fifth cycle. This steep decline indicates inadequate stability. The comparison demonstrates that SnS/SnO_*x*_–GO–PVP NC successfully retains active sites. The SEM analysis (Fig. S6) of SnS/SnO_*x*_–GO–PVP NC after five runs confirms that its structure remains intact, and the PXRD (Fig. S7) pattern of the reused SnS/SnO_*x*_–GO–PVP is similar to that of the fresh nanocomposite. Also, the FTIR data of the recycled catalyst are similar to the pristine material (Fig. S8). Thus, the results indicate the stability and reusability of the catalyst.

## 5. Conclusion

The SnS/SnO_*x*_ nanoparticle and SnS/SnO_*x*_–GO–PVP nanocomposite have been successfully synthesized using a hydrothermal technique. SnS/SnO_*x*_ NPs and SnS/SnO_*x*_–GO–PVP NC retain the Sn–S vibration (615–629 cm^−1^) and display additional carbonyl, C, N, and oxygenated groups that enable multivalent methylene blue adsorption through hydrogen bonding, coordination to PVP, π–π stacking with GO, and electrostatic attraction, as confirmed by FTIR spectroscopy and XRD. The thermogravimetric study shows increased thermal stability, with the NC remaining intact up to around 543 °C, whereas SnS/SnO_*x*_ NPs degrade at lower temperatures. X-ray photoelectron spectroscopy confirms the presence of C, N, O, S, and Sn, as well as the presence of Sn^2+^/Sn^4+^ oxides, partly oxidised GO, and PVP-derived nitrogen coordinating to Sn sites, indicating full component integration. The NC effectively eliminates ∼98% of MB (4–10 ppm) within 100 min, outperforming SnS/SnO_*x*_ NPs. The SnS/SnO_*x*_–GO–PVP nanocomposite emerges as an efficient catalyst for the selective conversion of nitrobenzene derivatives to the corresponding azobenzenes (70–83%) and further enables the selective oxidation of primary and secondary benzylic alcohols to the corresponding aldehydes and ketones in good to excellent yields (75–87%). Furthermore, the catalyst demonstrates good structural stability and retains its adsorption and catalytic efficacy for up to four and five consecutive reaction cycles, respectively. Notably, the narrow band gap and strong visible-light absorption of SnS impart intrinsic photocatalytic activity, thereby facilitating efficient charge generation and transfer within the hybrid architecture. Combined with the conductive graphene oxide network and biocompatible PVP matrix, this synergistic design enhances redox efficiency and environmental benignity, underscoring the material's potential for sustainable photo-assisted catalytic applications. These findings show that SnS/SnO_*x*_–GO–PVP is a scalable and cost-effective platform for combined wastewater treatment and selective organic transformations.

## Author contributions

Ashlesha P. Kawale: methodology, software, investigation, formal analysis, data curation, writing – original draft preparation, conceptualization; Nishant Shekhar: methodology, data curation, software, writing – original draft preparation; Kumari Anchal: writing – original draft preparation, data curation, formal analysis, investigation; S. Y. Bodkhe: data curation, formal analysis, investigation, supervision; Subhash Banerjee: supervision, resources, validation, writing – reviewing and editing; Arti Srivastava: conceptualization, methodology, supervision, resources, investigation, formal analysis, validation, writing – reviewing and editing, project administration, funding acquisition.

## Conflicts of interest

The authors declare that they have no known competing financial interests or personal relationships that could have appeared to influence the work reported in this paper.

## Supplementary Material

RA-016-D6RA02696F-s001

## Data Availability

The data supporting the findings of this study are available within the article and its supplementary information (SI). Supplementary information is available. See DOI: https://doi.org/10.1039/d6ra02696f.
