# Supplementary material for: Interfacial SnS/SnOx heterostructures on GO–PVP enable the removal of methylene blue and enhanced redox catalysis
Source: RSC Adv. 2026 Jul 6;16(36):38080–96. doi: 10.1039/d6ra02696f (PMC13335516; doi:10.1039/d6ra02696f)
Supplement: Supplementary file 1 [file RA-016-D6RA02696F-s001.pdf]

Supplementary Information (SI) for RSC Advances.
This journal is © The Royal Society of Chemistry 2026

Interfacial SnS/SnO_X Heterostructures on GO-PVP Enable Removal of Methylene Blue and Enhanced Redox Catalysis

Ashlesha P. Kawale[a,c], Nishant Shekhar[a,b], Kumari Anchal[a], S.Y. Bodkhe[c], Subhash Banerjee[a*] and Arti Srivastava[a*]

[a]*Department of Chemistry, Guru Ghasidas Vishwavidyalaya, Koni, Bilaspur - 495009, CG, India,*
[b]*Department of Chemistry, Institute of Science, Banaras Hindu University, Varanasi-221005, UP, India*
[c]*National Environmental Engineering Research Institute, NEERI, Nagpur - 440020, MH, India*
Correspondence author email: Arti Srivastava[a*] reach2arti@yahoo.co.uk

S. No.	Section	Table of Contents	Page No.
1	Figure S1	TGA thermogram of SnS/SnO_X NPs and SnS/SnO_X-GO-PVP NC	S2
2	Table S1	BET experimental results of SnS/SnO_X NPs and SnS/SnO_X-GO-PVP NC before adsorption	S2
3	Table S2	BET experimental results of SnS NPs and SnS/SnO_X-GO-PVP NC after adsorption	S3
4	Table S3	The % removal of MB dye by SnS/SnO_X NPs and SnS/SnO_X-GO-PVP NC	S3
5	Table S4	The % removal of dye by SnS/SnO_X NPs and SnS/SnO_X-GO-PVP NC after 100 minutes	S3
6	Figure S2	Crude High Resolution Mass Spectra data of the reaction mixture obtained from the reduction of Nitrobenzene	S4
7	Figure S3	Copies of ^{1}H NMR and ^{13}C NMR spectra of azobenzene	S5
8	Figure S4	Copies of ^{1}H NMR spectra of the products listed in Table 4	S6-S7
9	Figure S5	Copies of ^{1}H NMR and ^{13}C NMR spectra of the products listed in Table 6	S8-S10
10	Figure S6	SEM image of recycled SnS/SnO_X-GO-PVP nanocomposite	S11
11	Figure S7	Powder XRD of SnS/SnO_X-GO-PVP nanocomposite	S11
12	Figure S8	FTIR of recycled SnS/SnOx-GO-PVP NC	S12
13	Figure S9	Pseudo-first order kinetics	S12
14	Cal. S1	Calculation of the relative proportions of Sn^{2+}/Sn^{4+} from XPS	S13
15	Cal. S2	Calculation of the relative proportions of Sn^{2+}-O/Sn^{4+}-O from XPS	S13

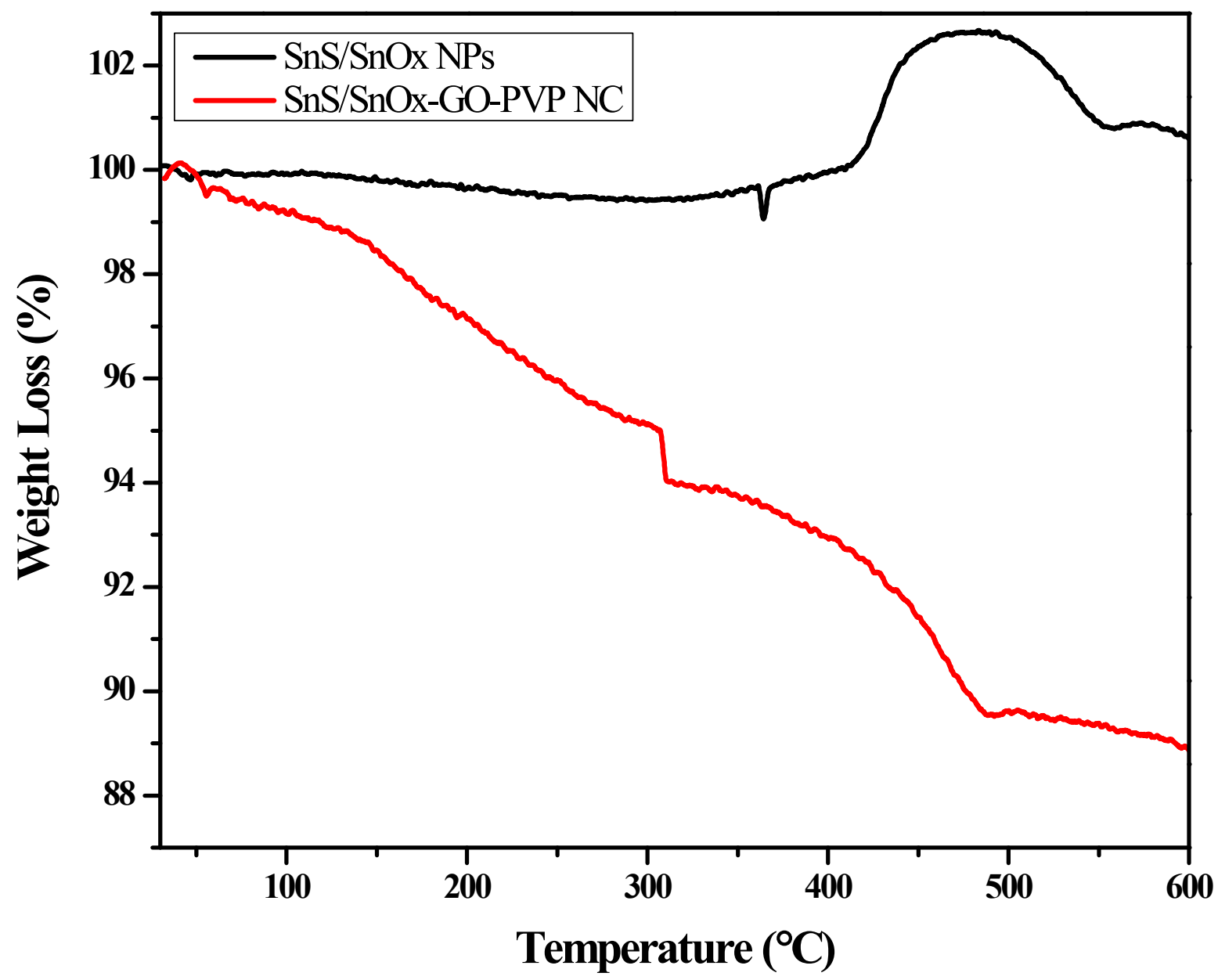

Fig. S1. TGA thermogram of SnS/SnO_X Nps and SnS/SnO_X-GO-PVP NC

Table S1. BET experimental results of SnS/SnO_X Nps and SnS/SnO_X-GO-PVP NC before adsorption

Sample	BJH adsorption (Before adsorption)			BJH desorption (Before adsorption)		
	Surface area (m^2/g)	Pore Volume (cc/g)	Pore Radius (A°)	Surface area (m^2/g)	Pore Volume (cc/g)	Pore Radius (A°)
SnS Nps	6.626	0.016	19.096	8.074	0.014	15.270
SnS/SnO_X-GO-PVP NC	5.502	0.014	15.279	5.288	0.013	17.056

Table S2. BET experimental results of SnS/SnO_X Nps and SnS/SnO_X-GO-PVP NC after adsorption

Sample	BJH adsorption (After adsorption)			BJH desorption (After adsorption)		
	Surface area (m^2/g)	Pore Volume (cc/g)	Pore Radius (A°)	Surface area (m^2/g)	Pore Volume (cc/g)	Pore Radius (A°)
SnS Nps	3.246	0.011	19.152	3.331	0.011	19.103
SnS/SnO_X-GO-PVP NC	8.375	0.045	15.285	8.545	0.045	17.051

Table S3. The % removal of MB dye by SnS Nps and SnS/SnO_X-GO-PVP NC

Dye Concentration(ppm)	% Removal of dye by SnS/SnO_X Nps	% Removal of dye by SnS/SnO_X-GO-PVP NC
4	89.83	98.78
6	88.23	97.95
8	86.06	96.1
10	82.67	95.01

Table S4. The % removal of dye by SnS/SnO_X Nps and SnS/SnO_X-GO-PVP NC after 100 minutes

Dye Concentration (ppm)	% Removal of dye by SnS/SnO_X Nps after 100 minutes	% Removal of dye SnS/SnO_X-GO-PVP NC after 100mins
4	89.66	98.71
6	88.24	97.93
8	86.26	96.08
10	82.56	94.90

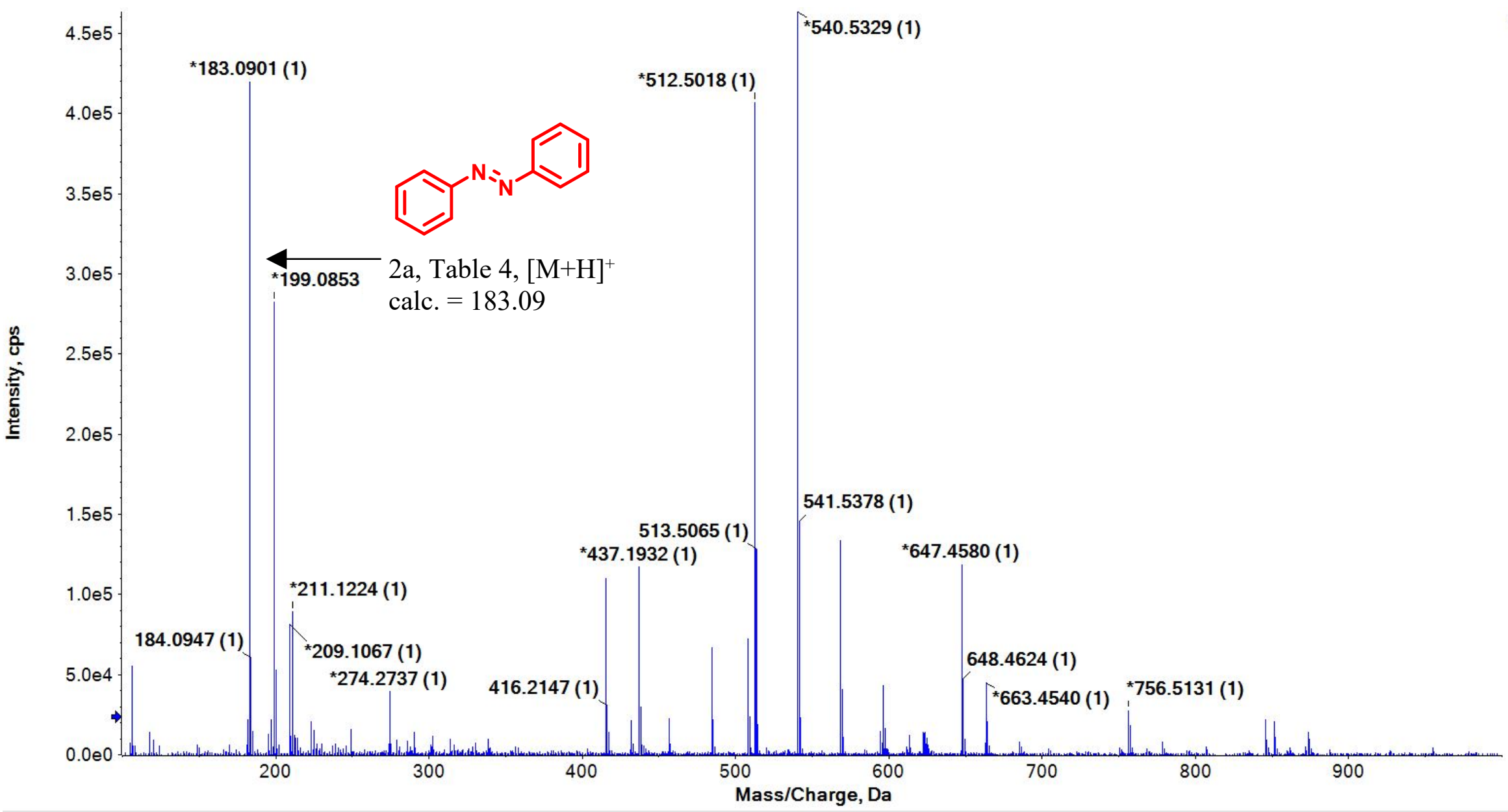

Fig. S2. Crude High Resolution Mass Spectra data of the reaction mixture obtained from the reduction of nitrobenzene

Fig. S3. Copies of ^{1}H NMR and ^{13}C NMR spectra of azobenzene

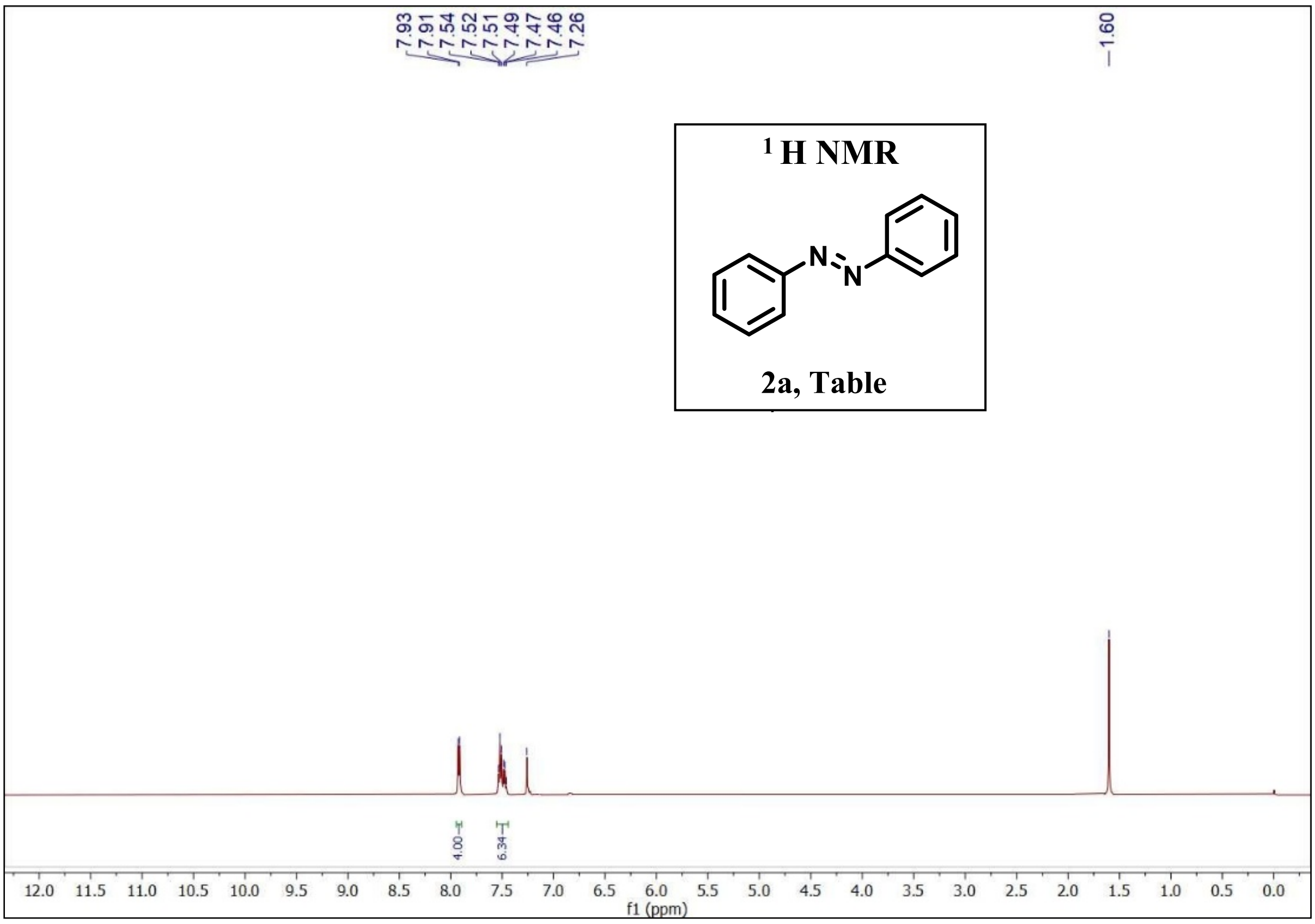

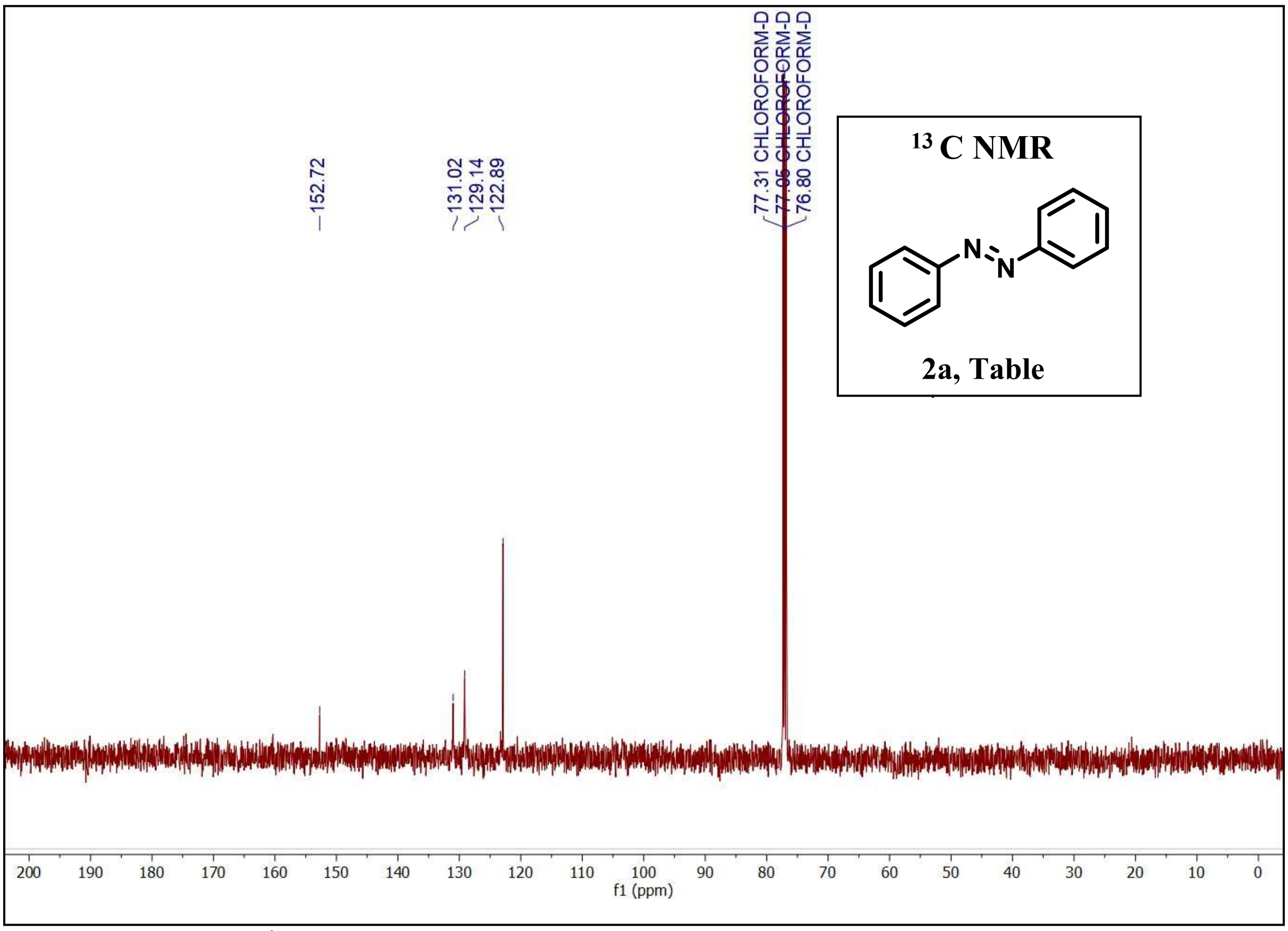

Fig. S4. Copies of ^{1}H NMR spectra of products listed in Table 4

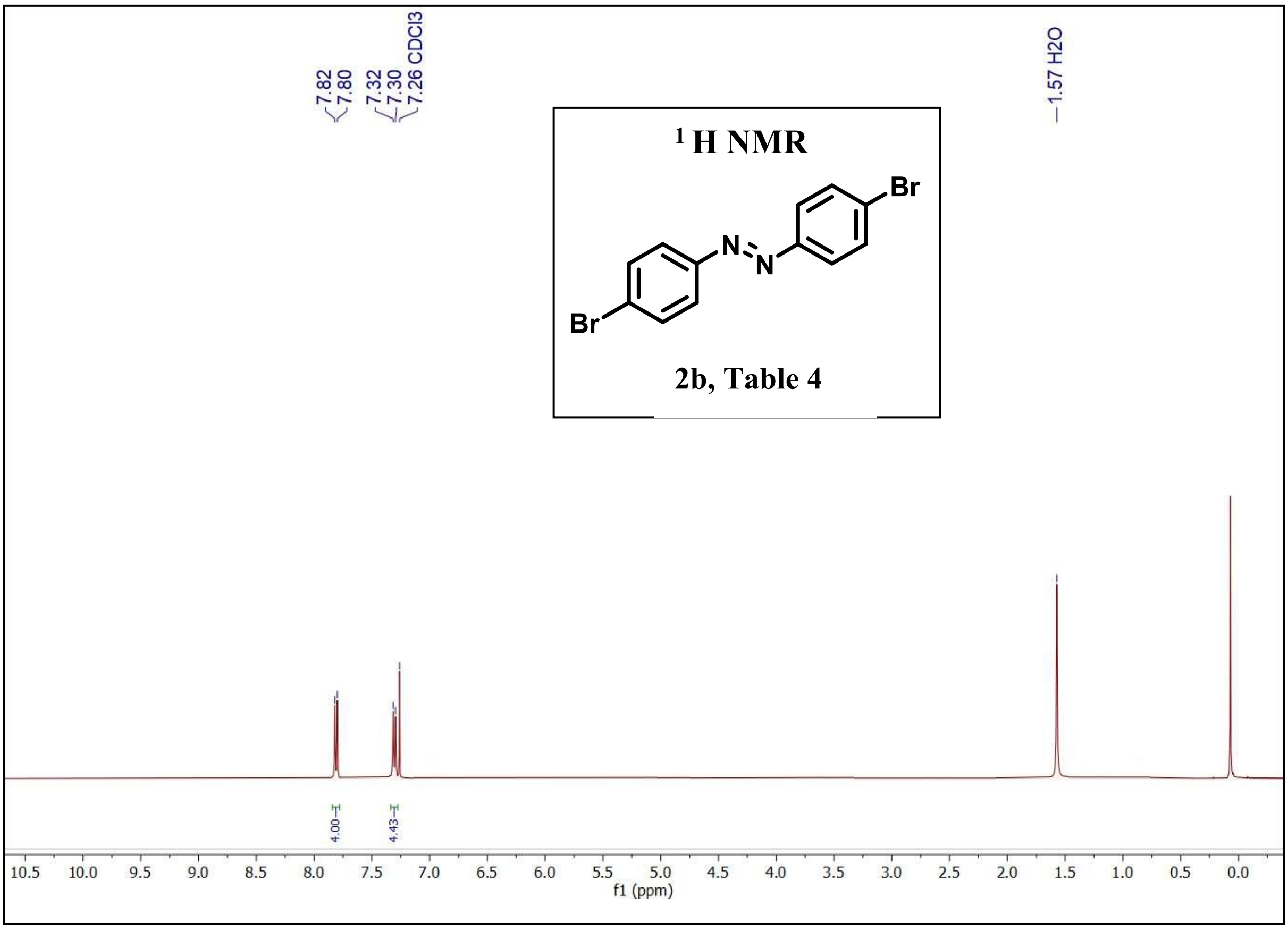

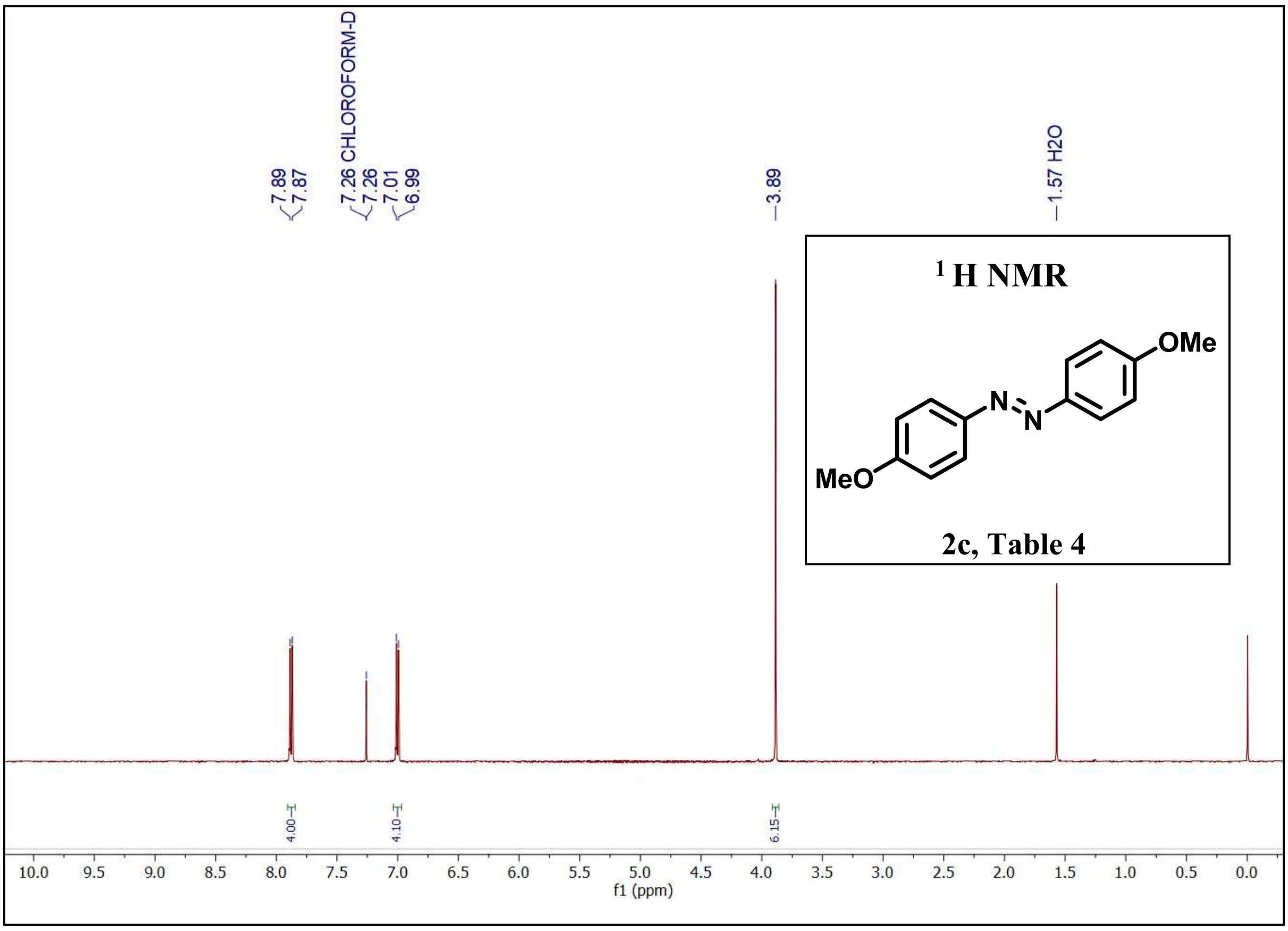
7.89
7.87
7.26 CHLOROFORM-D
7.26
7.01
6.99
3.89
1.57 H2O
1 H NMR
OMe
N
N
MeO
2c, Table 4
4.00
4.10
6.15
10.0 9.5 9.0 8.5 8.0 7.5 7.0 6.5 6.0 5.5 5.0 4.5 4.0 3.5 3.0 2.5 2.0 1.5 1.0 0.5 0.0
f1 (ppm)

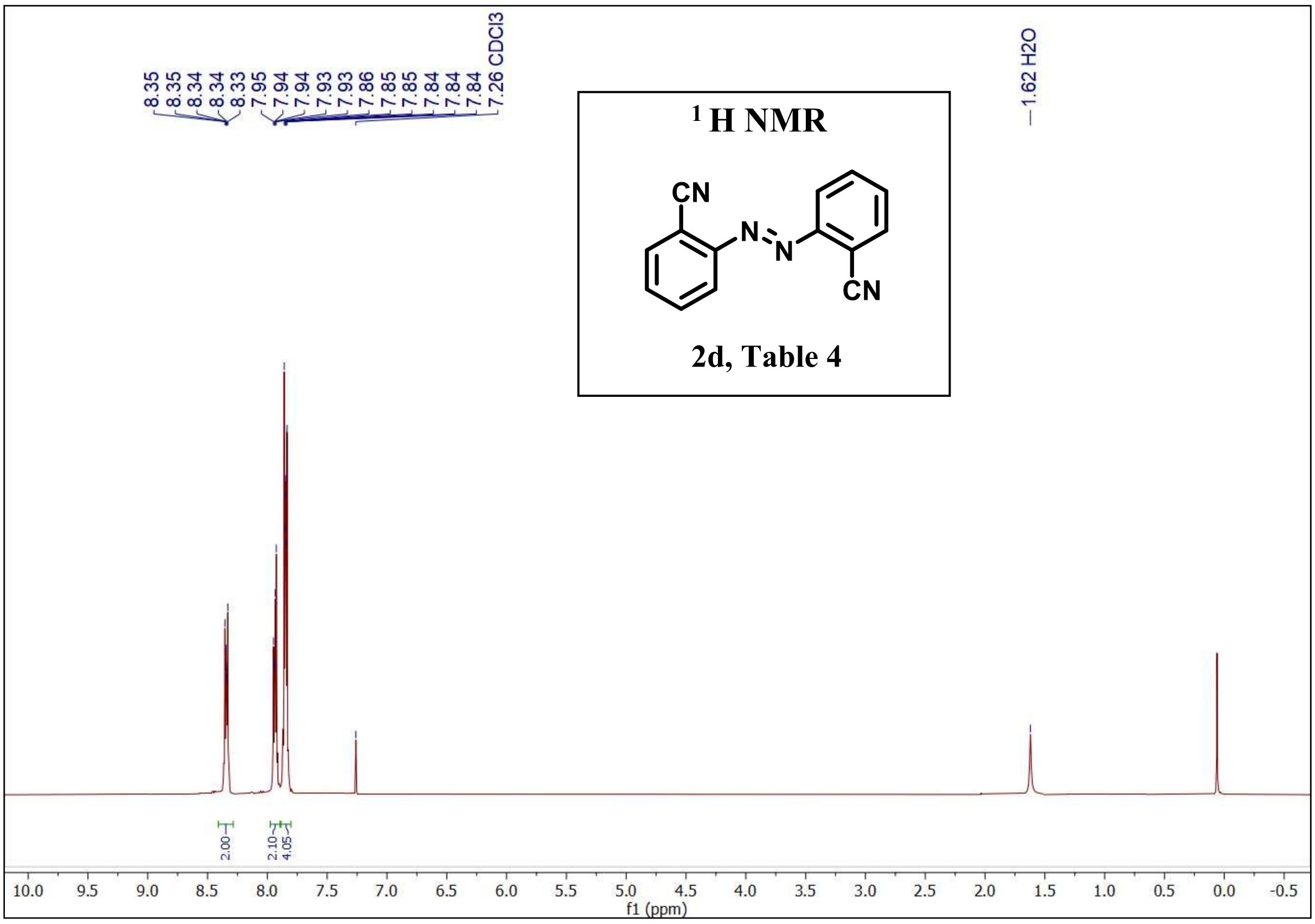
8.35
8.35
8.34
8.34
8.33
7.95
7.94
7.94
7.93
7.93
7.86
7.85
7.85
7.84
7.84
7.84
7.26 CDCl3
1.62 H2O
1 H NMR
CN
N
N
CN
2d, Table 4
2.00
2.10
4.05
10.0 9.5 9.0 8.5 8.0 7.5 7.0 6.5 6.0 5.5 5.0 4.5 4.0 3.5 3.0 2.5 2.0 1.5 1.0 0.5 0.0 -0.5
f1 (ppm)

Fig. S5. Copies of ^{1}H NMR and ^{13}C NMR spectra of the products listed in Table 6

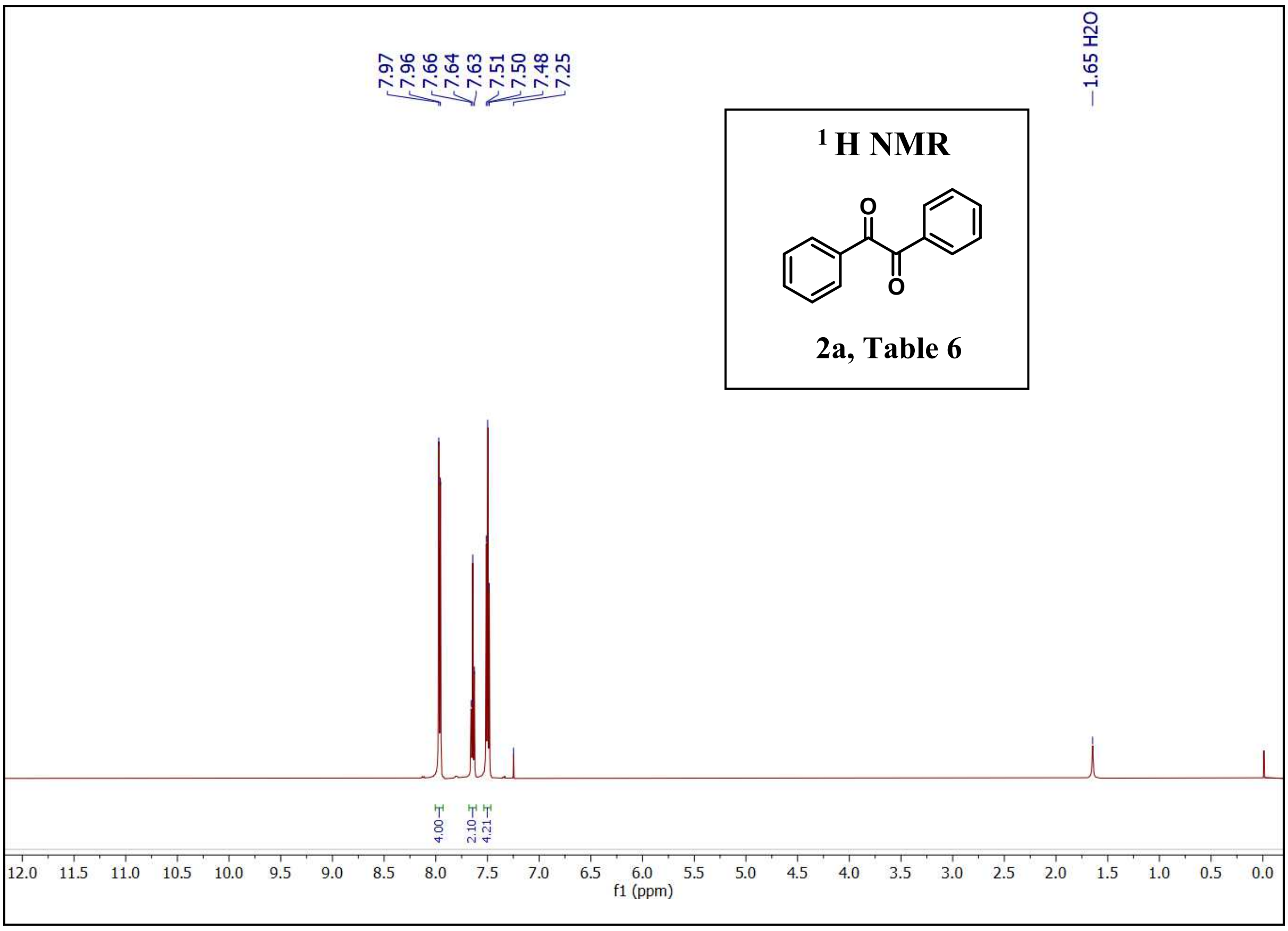

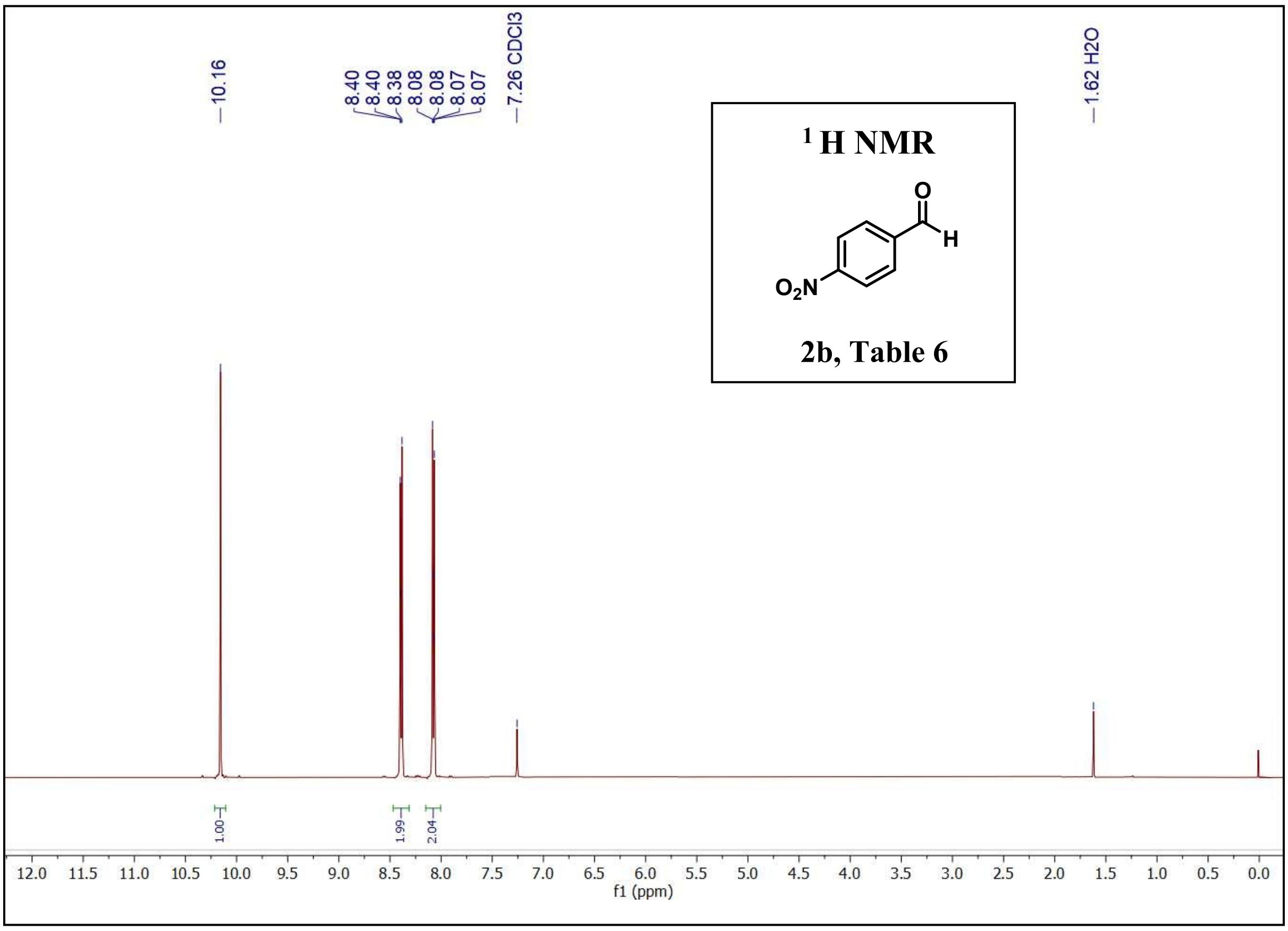

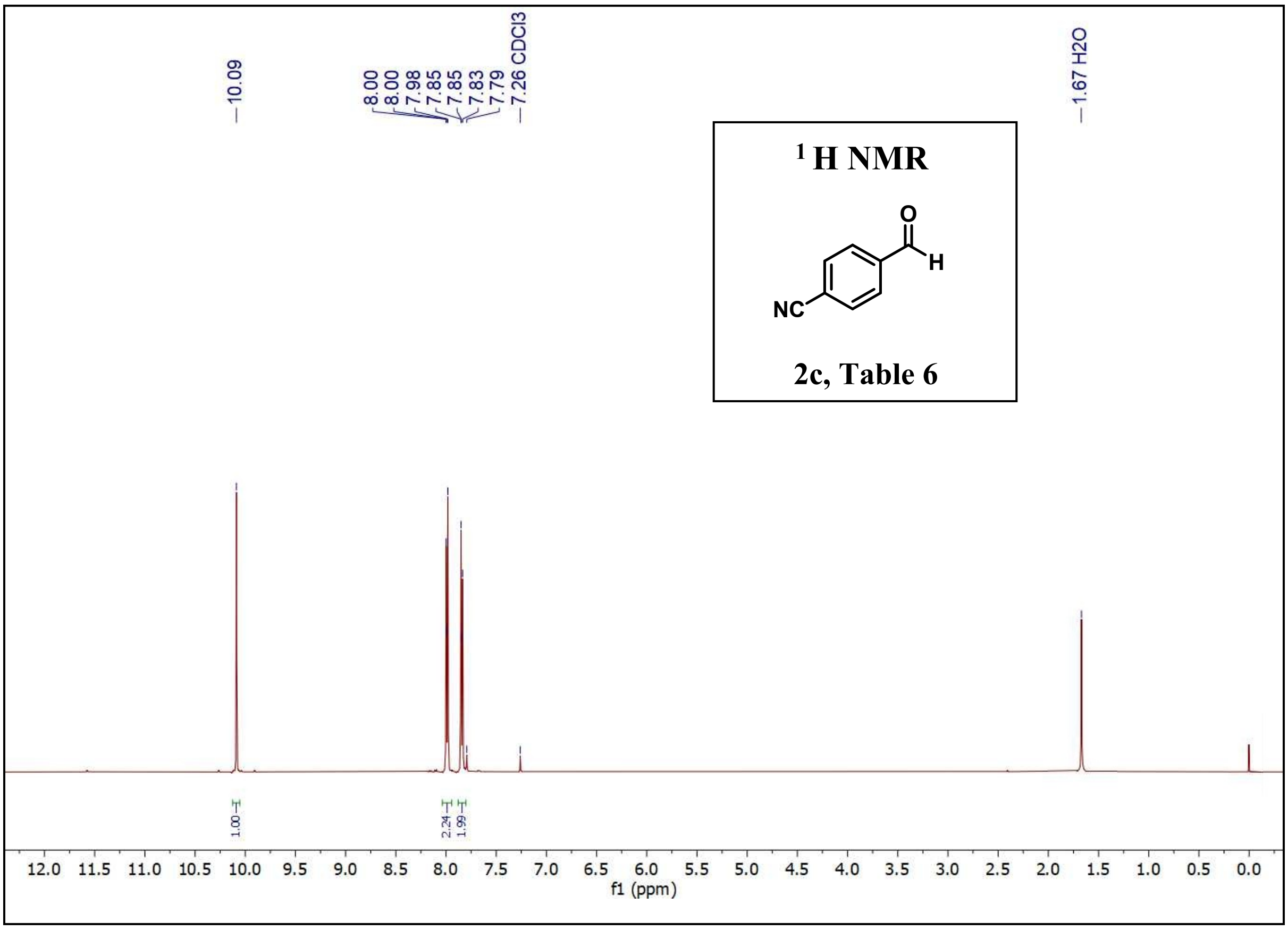

10.09
8.00
8.00
7.98
7.85
7.85
7.83
7.79
7.26 CDCl3
1.67 H2O
1 H NMR
O
H
NC
2c, Table 6
1.00
2.24
1.99
12.0 11.5 11.0 10.5 10.0 9.5 9.0 8.5 8.0 7.5 7.0 6.5 6.0 5.5 5.0 4.5 4.0 3.5 3.0 2.5 2.0 1.5 1.0 0.5 0.0
f1 (ppm)

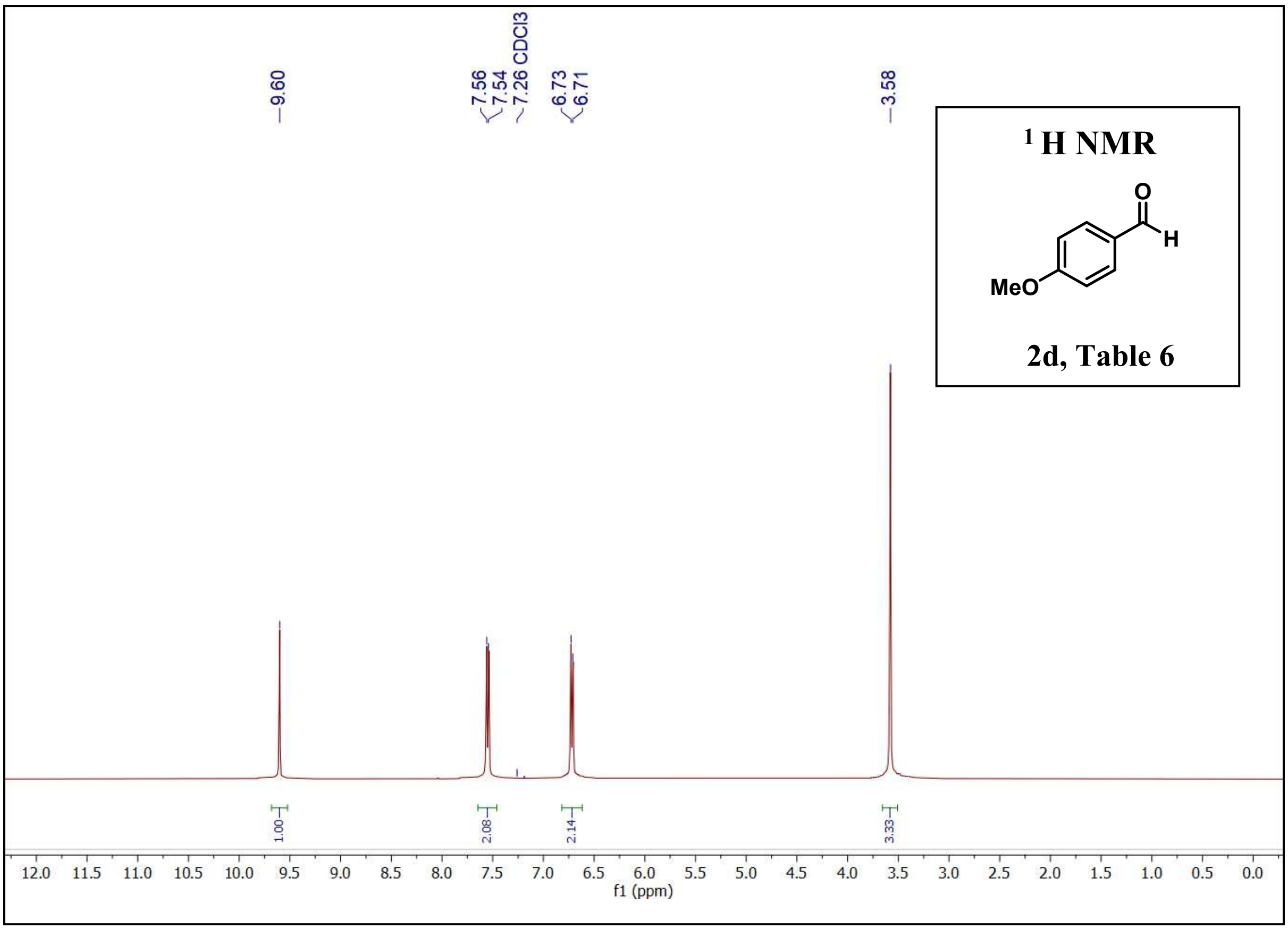

9.60
7.56
7.54
7.26 CDCl3
6.73
6.71
3.58
1 H NMR
O
H
MeO
2d, Table 6
1.00
2.08
2.14
3.33
12.0 11.5 11.0 10.5 10.0 9.5 9.0 8.5 8.0 7.5 7.0 6.5 6.0 5.5 5.0 4.5 4.0 3.5 3.0 2.5 2.0 1.5 1.0 0.5 0.0
f1 (ppm)

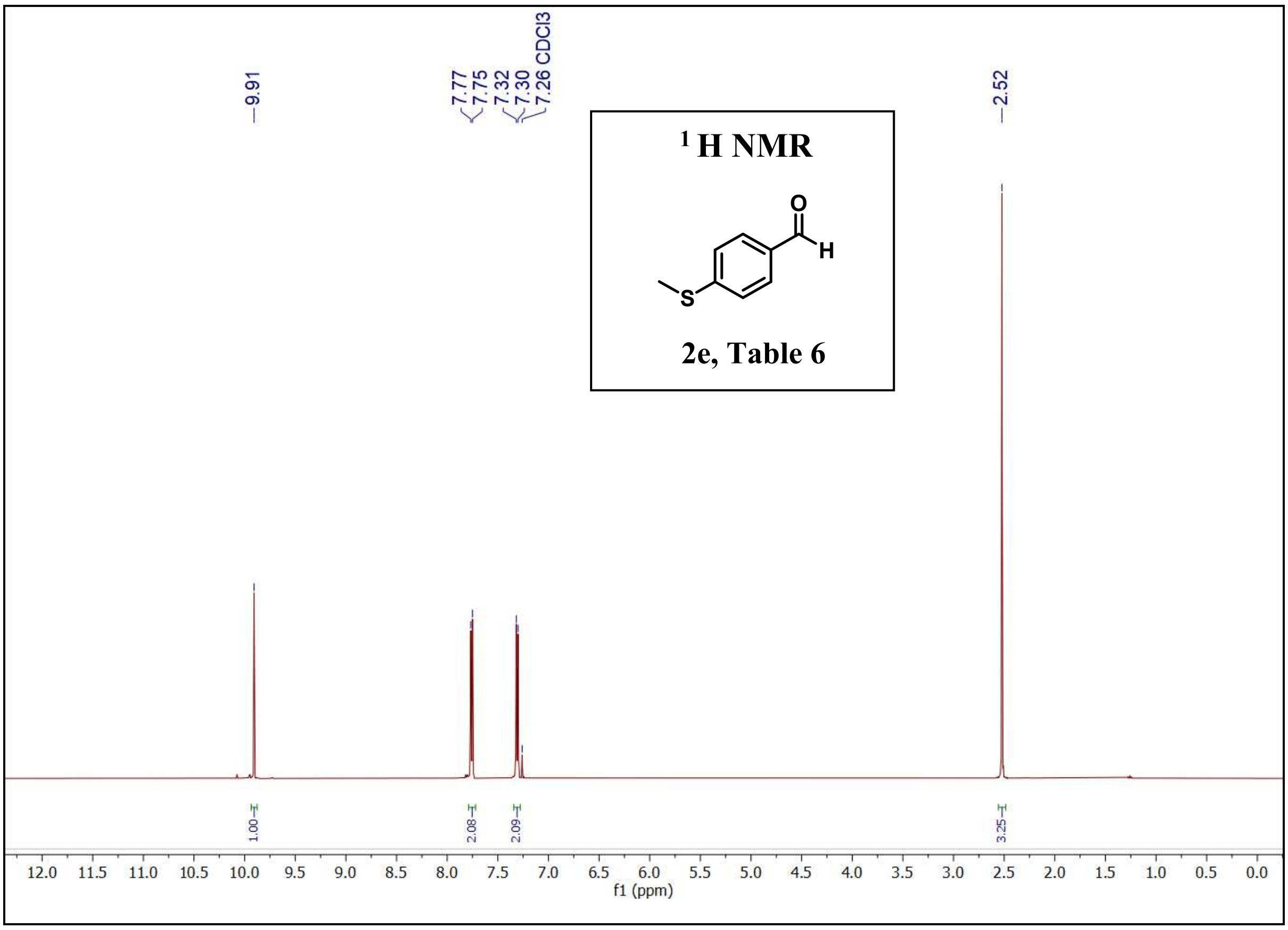
9.91
7.77
7.75
7.32
7.30
7.26 CDCl3
2.52
1 H NMR
O
H
S
2e, Table 6
1.00
2.08
2.09
3.25
12.0 11.5 11.0 10.5 10.0 9.5 9.0 8.5 8.0 7.5 7.0 6.5 6.0 5.5 5.0 4.5 4.0 3.5 3.0 2.5 2.0 1.5 1.0 0.5 0.0
f1 (ppm)

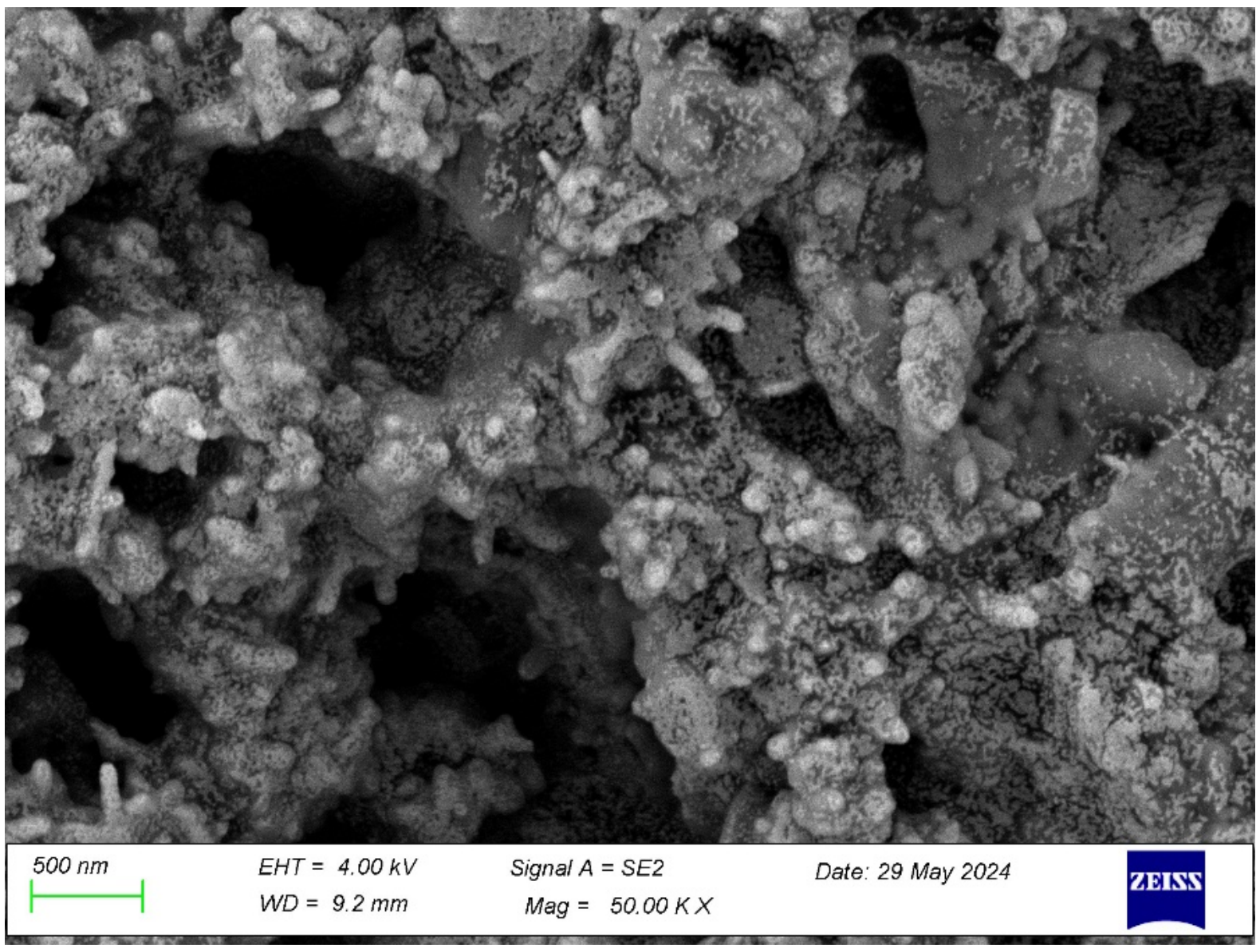

Fig. S6. SEM image of recycled SnS/SnO_X-GO-PVP nanocomposite

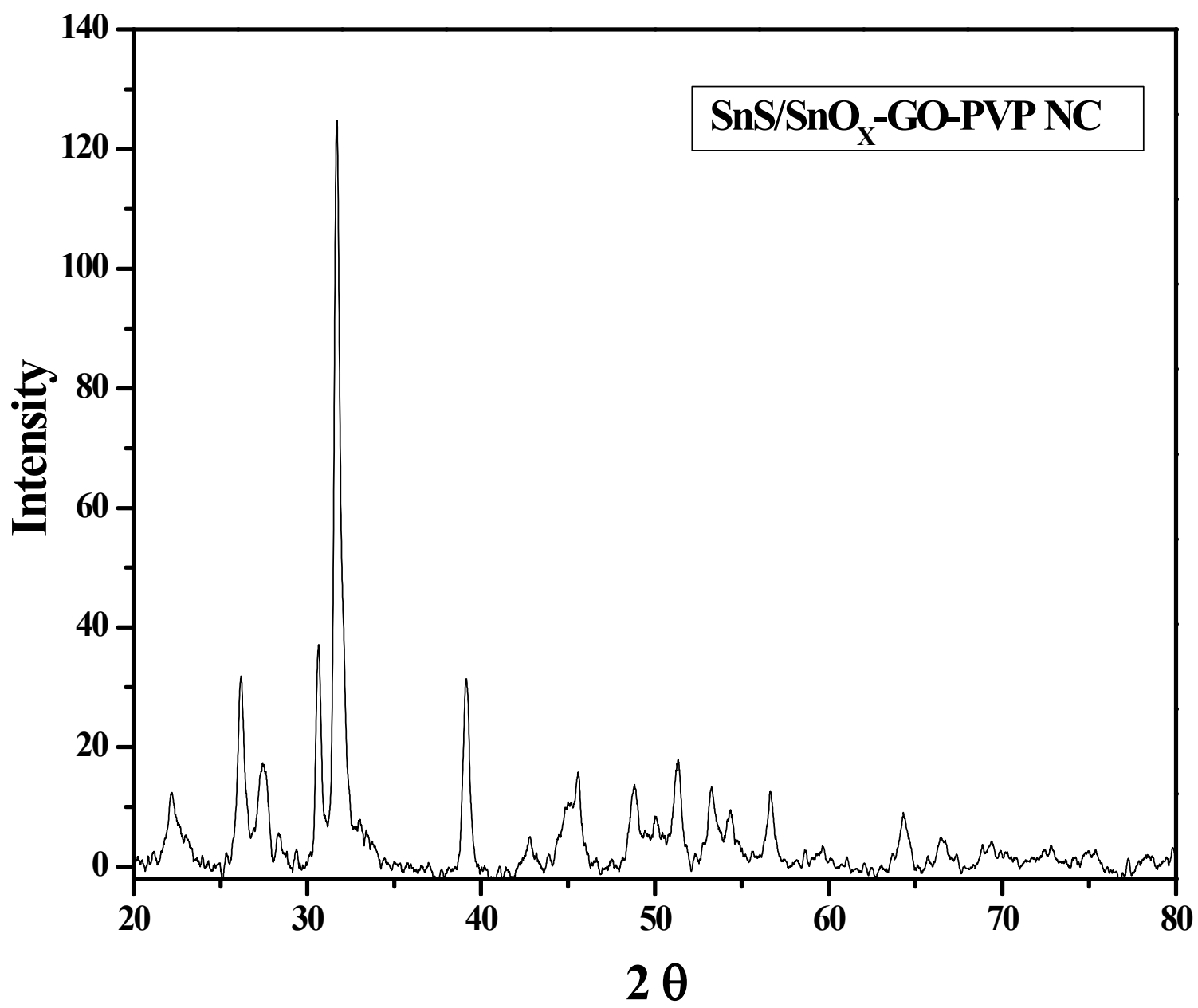

Fig. S7. Powder XRD of recycled SnS/SnO_X-GO-PVP nanocomposite

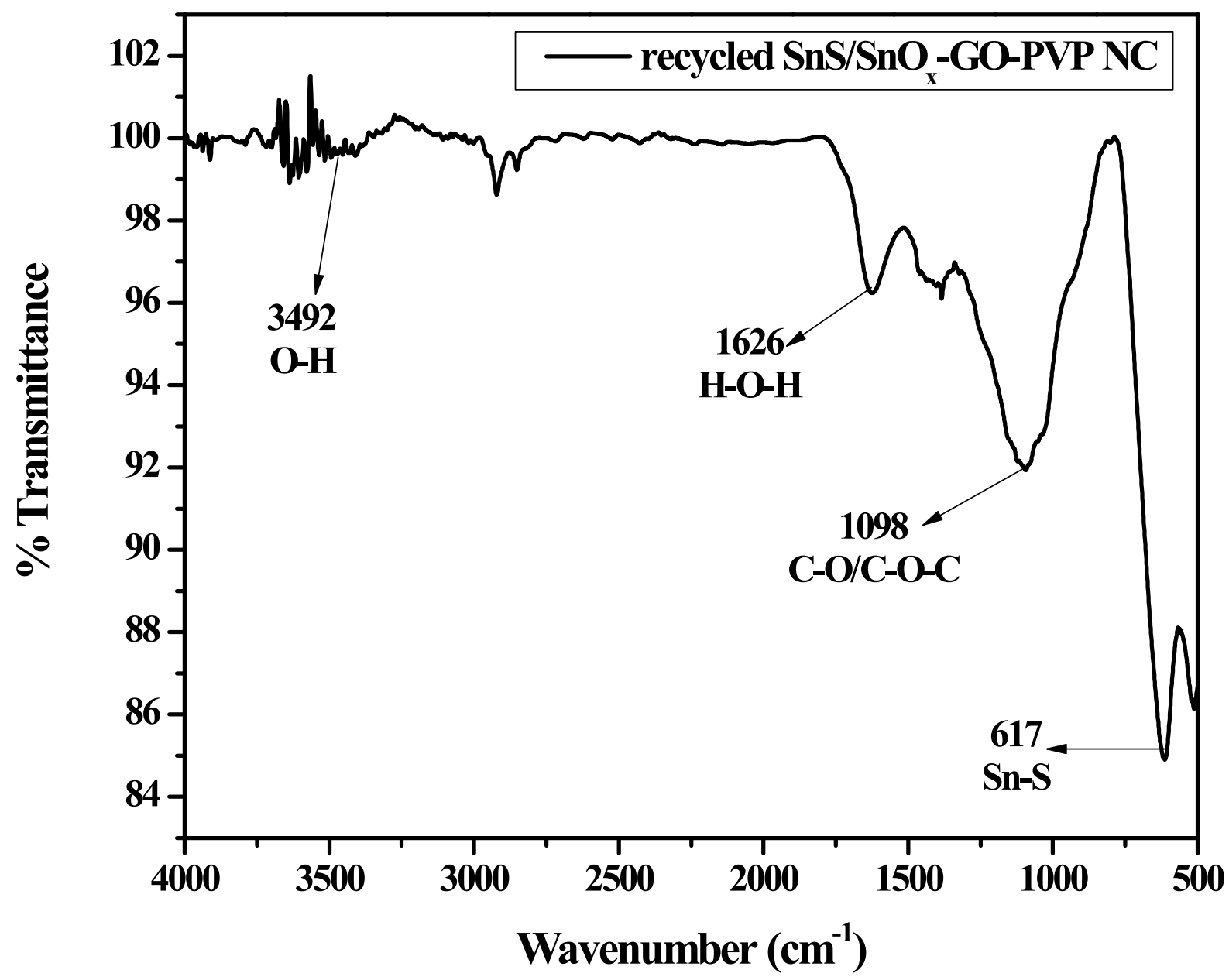

Fig. S8 FTIR of recycled SnS/SnOx-GO-PVP NC

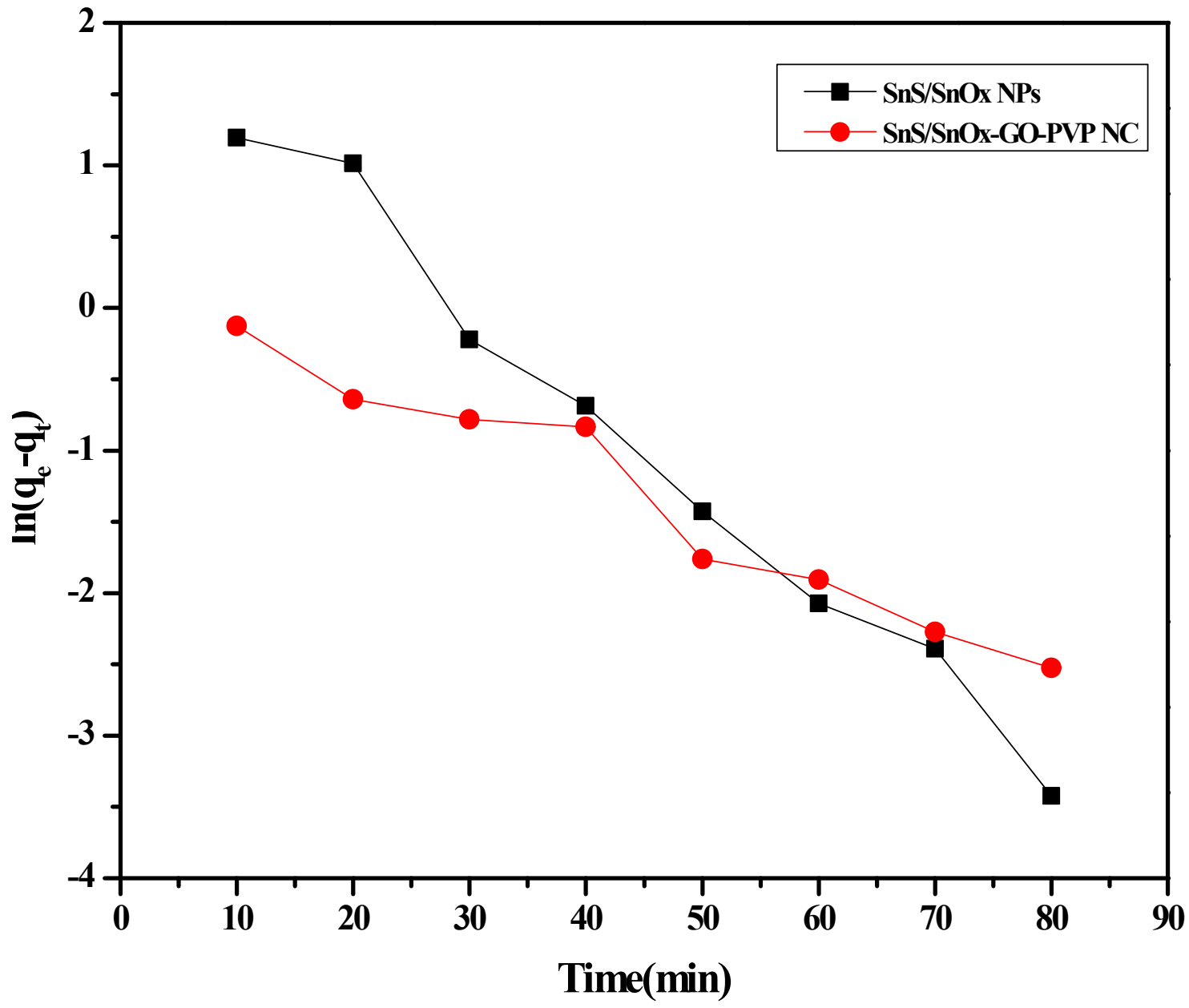

Fig. S9 Pseudo-first order kinetics

Total Sn^{2+} area (A_{Sn2+}) = $A_{3d5/2} + A_{3d3/2}$

= 69970.874025 + 69971.6298

= 139942.503825

Total Sn^{4+} area (A_{Sn4+}) = $A_{3d5/2} + A_{3d3/2}$

= 69971.61 + 69971.6286

= 139943.2386

Total Sn area = $A_{Total} = A_{Sn2+} + A_{Sn4+}$

= 139942.503825 + 139942.503825

= 279885.742425

Sn^{2+} percentage = Sn^{2+} (%) = $\frac{139942.503825}{279885.742425} \times 100$

= 49.999%

Sn^{4+} percentage = Sn^{4+}(%) = $\frac{139943.2386}{279885.742425} \times 100$

= 50.0001%

Therefore, the relative proportions of Sn^{2+}/Sn^{4+} were found to be 1:1.

Cal. S1 Calculation of the relative proportions of Sn^{2+}/Sn^{4+} from XPS

Total O 1s area = $A_{Total} = A_{Sn2+-O} + A_{Sn4+-O}$

= 71413.43035 + 67743.431675

= 139156.862025

Lattice oxygen % = $\frac{71413.43035}{139156.862025} \times 100$

= 51.32 %

Defect/oxygen-vacancy related oxygen % = $\frac{67743.431675}{139156.862025} \times 100$

= 48.68%

Cal. S2 Calculation of the relative proportions of Sn^{2+}-O/Sn^{4+}-O from XPS
